# Phylogenetic analysis of F-bZIP transcription factors indicates conservation of the zinc deficiency response across land plants

**DOI:** 10.1038/s41598-017-03903-6

**Published:** 2017-06-19

**Authors:** Pedro Humberto Castro, Grmay H. Lilay, Antonio Muñoz-Mérida, Jan K. Schjoerring, Herlânder Azevedo, Ana G. L. Assunção

**Affiliations:** 1 Department of Plant and Environmental Sciences, University of Copenhagen, Thorvaldsensvej 40, DK-1871 Copenhagen, Denmark; 2 CIBIO, InBIO - Research Network in Biodiversity and Evolutionary Biology, University of Porto, Campus Agrário de Vairão, 4485-661, Vairão, Portugal; 3 Department of Biology, Faculty of Sciences, University of Porto, Rua Campo Alegre, 4169-007, Porto, Portugal

## Abstract

Basic leucine zipper (bZIP) transcription factors control important developmental and physiological processes in plants. In *Arabidopsis thaliana*, the three gene F-bZIP subfamily has been associated with zinc deficiency and salt stress response. Benefiting from the present abundance of plant genomic data, we performed an evolutionary and structural characterization of plant F-bZIPs. We observed divergence during seed plant evolution, into two groups and inferred different selective pressures for each. Group 1 contains AtbZIP19 and AtbZIP23 and appears more conserved, whereas Group 2, containing AtbZIP24, is more prone to gene loss and expansion events. Transcriptomic and experimental data reinforced AtbZIP19/23 as pivotal regulators of the zinc deficiency response, mostly via the activation of genes from the ZIP metal transporter family, and revealed that they are the main regulatory switch of *AtZIP4*. A survey of *AtZIP4* orthologs promoters across different plant taxa revealed an enrichment of the *Zinc Deficiency Response Element* (*ZDRE*) to which both AtbZIP19/23 bind. Overall, our results indicate that while the AtbZIP24 function in the regulation of the salt stress response may be the result of neo-functionalization, the AtbZIP19/23 function in the regulation of the zinc deficiency response may be conserved in land plants (Embryophytes).

## Introduction

Regulatory networks and modulation of gene expression by transcription factors (TFs) provide cells and organisms with a dynamic and broad response to developmental and environmental cues. TFs can be grouped into different gene families according to the type of DNA-binding and multimerization domains they encode^1–3^. Basic leucine zipper (bZIP) proteins form a large family of TFs present in all eukaryotes. They bind to DNA as dimers and are characterized by a highly conserved 40- to 80-amino acid-long domain, the bZIP domain, that is composed of two motifs: a basic region involved in specific DNA binding, and a leucine-zipper domain that directs protein–protein interaction^4–6^. bZIP proteins can form homodimers and heterodimers, with dimerization specificity, variable dimer combinations, and post-translational regulation influence of *in vivo* dimer composition, all contributing to the diversity and flexibility of bZIP transcriptional regulation^7–9^.

Increasing availability of genomic information is showing that in plants a significant proportion of protein-coding genes are regulatory proteins. Taking Arabidopsis as an example, 6% of its ca. 27 thousand protein-coding genes are TFs. From an evolutionary perspective, it has been observed that several TF families have higher rates of expansion in plants than in other eukaryotes (e.g. bZIP, MADS, bHLH and MYB gene families)^10–12^. This can be partially explained by events of whole genome duplications (WGD) and small-scale genome duplications (SSD), which are more frequent in plants compared to other eukaryotes^13–15^. These gene duplication events have been acknowledged as a major source of evolutionary novelty. In addition, the understanding of the functional fate of duplicates and the generation of functional diversity, though being an active field of research, is suggested to depend on the type of duplication mechanism (WGD vs SSD), with gene dosage/stoichiometric balance constraints, and likely influenced by the plant species and its ecological requirements^14, 16–20^. Nonetheless, there is evidence that regulatory genes are preferentially retained, suggesting important roles of TF duplicates in plant evolution. A larger repertoire of TF duplicates in plants might play a significant role in the generation of new traits and colonization of new environments ultimately contributing to the remarkable adaptation of plants, as sessile organisms, to a wide range of ecological niches and a changing environment^11, 19, 21^.

The bZIP TF family, as mentioned above, has been significantly expanded in the plant lineage, being one of the largest groups of TFs in plants^10, 22^. Phylogenetic analysis of bZIP genes from algae, mosses, ferns, gymnosperms and angiosperms revealed evolutionary relationships that suggest that the ancestor of green plants (Viridiplantae) possessed four bZIP founder genes, from which the family amplified and significantly diverged, generating traits that benefited the colonization of new habitats^23^. The *A. thaliana* genome encodes 75 predicted bZIP TFs which have been divided into ten subfamilies (named A to I, plus S) based on the sequence similarities of the bZIP domain, shared intron positions and other conserved motif similarities^24^. Several *A. thaliana* bZIPs have been functionally characterized and implicated in development, metabolism and stress responses, including photomorphogenesis, tissue differentiation, seed maturation and flower development, energy metabolism, hormone and sugar signalling, and pathogen defence^24–29^.

The three members of the small F subfamily of *A. thaliana* bZIPs are AtbZIP19, AtbZIP23 and AtbZIP24, and have been associated with the regulation of zinc deficiency and salt stress response^30–32^. In a comparative transcriptomic analysis in response to salt stress, AtbZIP24 was suggested to function as a negative transcriptional regulator of the salt stress acclimation response^30^. Further studies showed that AtbZIP24 repressed several transcripts with a known function in the plant tolerance to salt and osmotic stress, i.e., involved in cellular Na^+^ uptake and intracellular transport, and in the maintenance of ionic and osmotic balance under salt stress^31^. Subsequently, AtbZIP19 and AtbZIP23 were shown to play a pivotal role as positive transcriptional regulators of the zinc deficiency response^32^. They were identified, in a yeast-one-hybrid screening, to associate to promoter regions of the zinc deficiency-induced *AtZIP4* gene of the Zrt, Irt-like protein (ZIP) family of metal transporters. They seem to act partially redundantly and the double mutant *Atbzip19 Atbzip23* is hypersensitive to zinc deficiency. Furthermore, transcriptomic profiling of the mutant revealed deregulation of only a small set of genes, including genes associated with zinc homeostasis and comprising additional *AtZIP* genes. These were characterized by the presence of the *cis-*element *Zinc Deficiency Response Element* (*ZDRE*) in their promoter region, to which both bZIP19 and −23 proteins can bind^32, 33^. In another study, AtbZIP19 was identified in an *A. thaliana* T-DNA insertion mutant screen with the *Atbzip19* mutant exhibiting zinc deficiency hypersensitivity. A quantitative proteomics analysis identified proteins whose expression is affected by AtbZIP19, including AtZIP members^34^. Within the *A. thaliana* F subfamily, the AtbZIP19 and AtbZIP23 share higher amino acid sequence identity when compared to AtbZIP24. However, all three F bZIP proteins display a characteristic His/Cys-rich motif which is a signature of this bZIP subfamily^24, 35^.

The current functional information for *A. thaliana* F bZIPs, which associates them with zinc deficiency and salt stress responses, are of agricultural and economic significance. In addition, there is indication of conservation of AtbZIP19 and AtbZIP23 orthologs across land plants, with closely related genes being found in several species, including rice, poplar, soybean, but also gymnosperms and mosses^32^. Considering the remarkable recent developments in plant whole-genome sequencing and comparative genomic methods, the present study provides a phylogenetic analysis of the predicted F bZIP subfamily orthologs in land plants (Embryophytes). The aim is to elucidate the evolutionary trajectory of this small subfamily of TFs, and to help understand the functional diversification between AtbZIP19/23 and AtbZIP24. Finally, by investigating conservation and diversification in the F bZIP subfamily across plant species, this work provides a framework for the translation of functional information between *A. thaliana* and crops.

## Results

### Phylogenetic characterization of plant F subfamily bZIPs

**Figure 1:**
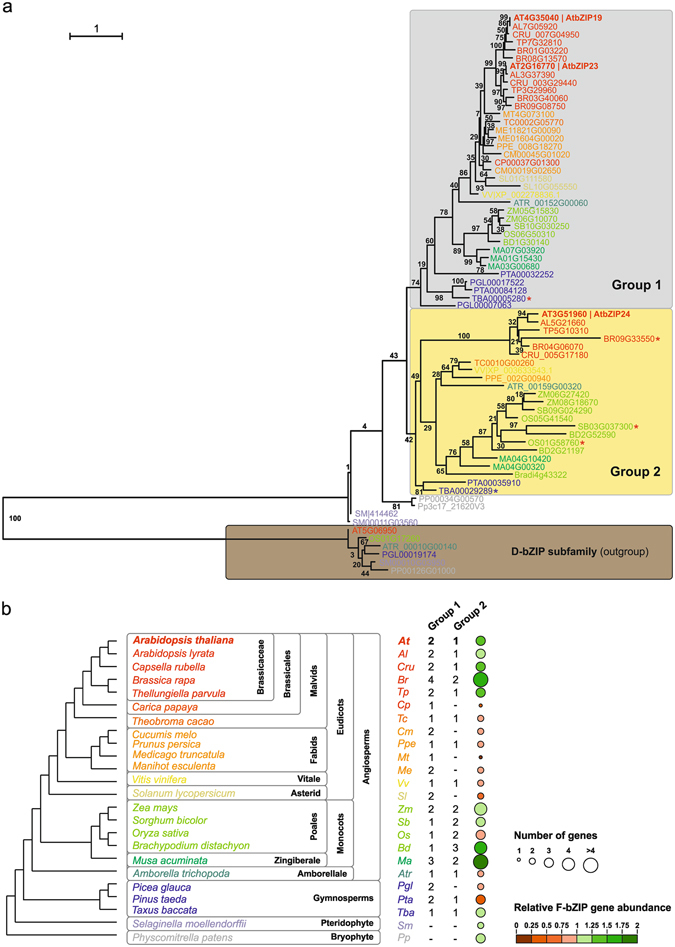
Phylogenetic analysis of the F-bZIP subfamily of proteins in plant species. **(a)** Proteins of the F-bZIP subfamily from a selection of 24 species representing all major plant taxa were used in the phylogenetic analysis. The phylogenetic tree was constructed using maximum-likehood and bootstrap values from 1000 replicates. Numbers on each branch represent the percentages of bootstrap. Proteins of the D-bZIP subfamily from representative species of different phylogenetic groups were used as an outgroup. Asterisks indicate proteins with missing or incomplete His/Cys-rich motif (red) or bZIP domain (blue). (**b**) F-bZIP gene enrichment for each plant species. Bubble size represents the absolute number of genes present in each species’ genome. Bubble colours indicate the relative size of the F-bZIP subfamily. The relative value was calculated as the ratio of F-bZIPs per total of genes in the genome versus the average of F-bZIPs in all species analysed.

In order to perform a comparative genomics characterization of the F subfamily of plant bZIP transcription factors (herein F-bZIP TFs), a set of 24 species was selected that represented all major plant taxa and emphasized angiosperm phylogeny. F-bZIP orthologs were retrieved using a strategy that combined web-based comparative genomics platforms and hand curation/BLAST analysis (Supplementary Fig. S1). No F-bZIPs were observed in the genomes of *Ostreococcus lucimarinus* and *Chlamydomonas reinhardtii*, representing green algae (Chlorophyta). In total, 67 F-bZIP sequences were identified (Supplementary Table S1). Gblocks was then used to infer on the presence of sequence integrity in the most conserved protein residues of F-bZIP subfamily members, resulting in the suppression of three gene entries for lack of the most conserved F-bZIP protein domains. Full protein sequences were subsequently used for phylogenetic inference (Fig. 1a). The outgroup consisted of one sequence per major taxon (Bryophytes, Pteridophytes, Gymnosperms, Monocots and Eudicots) of the D subfamily of plant bZIPs, which is reported to be the sister bZIP subgroup with closest evolutionary proximity to F-bZIPs^23, 24^. From a root containing genes from Bryophytes and Pteridophytes (*P. patens* and *S. moellendorffii*), we observed the branching out of two clades, each containing representatives of all major seed plant (Spermatophyta) taxa. These were subsequently named Group 1 and 2. Group 1 includes *A. thaliana bZIP19*/AT4G35040 and *bZIP23*/AT2G16770, and Group 2 includes *bZIP24*/AT3G51960. These results suggest a monophyletic origin for F-bZIPs with subsequent expansion during seed plant evolution.

The comparative genomics analysis shows that F-bZIPs are a small subfamily, comprising on average two to three members per species (Fig. 1b), with more F-bZIPs belonging to Group 1 than to Group 2 (i.e. 37 and 23, respectively). While all species have at least one TF in Group 1, not all species are represented in Group 2, which suggests independent gene loss events throughout Group 2 evolution (Fig. 1a,b). Interestingly, the species expansion/depletion plot suggests the occurrence of an F-bZIP enrichment in Monocots and Brassicaceae (Fig. 1b). Genes clustering closer to *AtbZIP19* and *AtbZIP23* seem to represent a Group 1 lineage-specific pair of paralogs, resulting from a gene duplication event associated with Brassicaceae. Conversely, Monocot enrichment seems related with Group 2 genes, in which all species display two to three Group 2 F-bZIPs.

### Functional insights from protein conservation, genomic synteny and gene expression patterns

**Figure 2:**
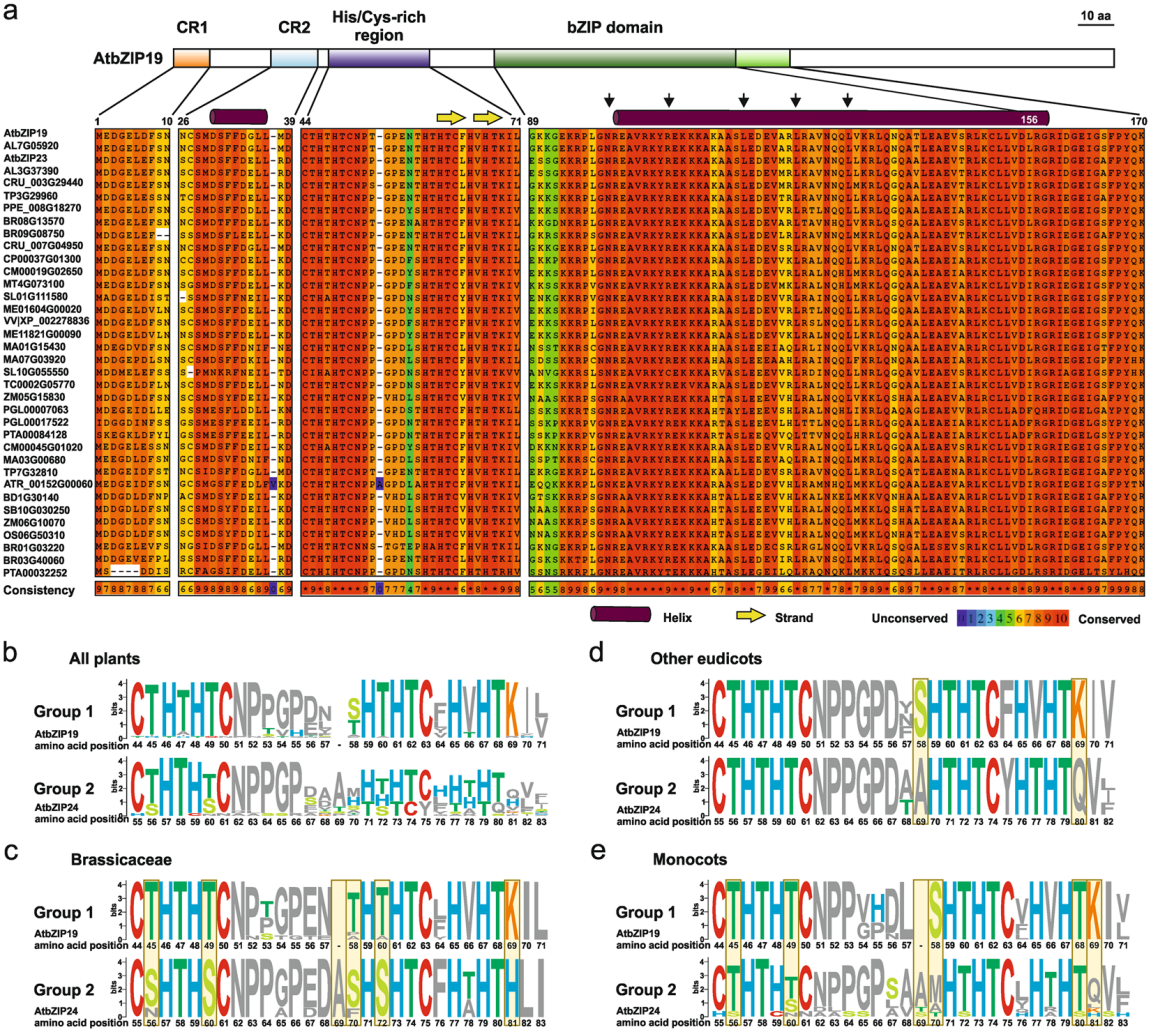
Conserved domain analysis of F-bZIP proteins. (**a**) Multiple sequence alignment of conserved regions matching significant protein motifs and domains of the Group 1 F-bZIPs. Amino acid consistency is classified from 0 (unconserved) to 10 (conserved); perfect amino acid match in all species is indicated with an asterisk. CR1/2 stands for conserved region 1 or 2. The AtbZIP19 was used as reference sequence. Secondary structure was predicted for AtbZIP19 and displayed on top of the alignment. Inset arrows represent highly conserved residues of the bZIP domain consensus sequence [N-X7-R/K-X9-L-X6-L-X6-L]. Sequence logo were used to compare the His/Cys-rich motif between F-bZIP Groups 1 and 2 in all plants. Four sequences with this motif missing or incomplete were not included (Fig. 1a) (**b**), Brassicaceae species **(c)**, other Eudicots (only the ones which also have an F-bZIP from group 2, *T. cacao*, *P. persica* and *V. vinifera*) (**d**), and Monocots (**e**). Letters denote amino acids and the height amplitudes indicate the degree of conservation for the amino acids in that position. The amino acid number position is in relation to AtbZIP19 for F-bZIP Group 1 and AtbZIP24 for F-bZIP Group 2 proteins. Colours highlight amino acids of interest. Yellow boxes highlight the most divergent amino acids between Groups 1 and 2.

Evolution of distinct F-bZIP groups in seed plants suggests structural differentiation at the protein level. To obtain further insight, we looked at protein sequence conservation via multiple sequence alignment (Fig. 2a, Supplementary Figs S2 and S3). F-bZIPs from Groups 1 and 2 show an overall high degree of conservation for the bZIP domain, containing its consensus sequence with highly conserved residues [N-X7-R/K-X9-L-X6-L-X6-L]. In addition, presence of a characteristic F-bZIP His/Cys-rich motif [C[ST]HTH[ST]CNP[PT]GPE-H[ST]HTC[FL]H[AV]HT]^24^ was also observed. From the total 64 bZIPs in the phylogenetic tree, five had an incomplete or missing His/Cys-rich motif or bZIP domain (Fig. 1a, Supplementary Figs S2 and S3). Considering the His/Cys-rich motif, a more detailed analysis with sequence logo representations showed an overall conservation of the histidine (H) and cysteine (C) residues in both F-bZIP groups (Fig. 2b). However, the motif in Group 2 is less conserved and shows more variations than in Group 1, partly due to an additional amino acid residue, most often an alanine (A), in Brassicaceae and Monocots from Group 2. This causes a shift, affecting the positional conservation of downstream H and C residues (Fig. 2c,d,e). In addition, Group 2 proteins of Brassicaceae have an additional and conserved H in position +81, and show substitutions from a threonine (T) to a serine (S) at positions +56, +60, +70 and +72, which appear less frequently in other families (Fig. 2c). Group 1 proteins, on the other hand, show a conserved lysine (K) residue in position + 69 which in Group 2 is only found in one Monocot sequence, BD2G21197 (Fig. 2b; Supplementary Fig. S3). The analysis identified two additional conserved regions. These are a 10-amino acid region at the protein N-terminus, and a 13-amino acid region just a few residues upstream of the His/Cys-rich motif, which were identified in sequences from Group 1. Structural prediction analysis in the 13-amino acid region predicts a helical structure (Fig. 2a, Supplementary Figs S2 and S3).

**Figure 3:**
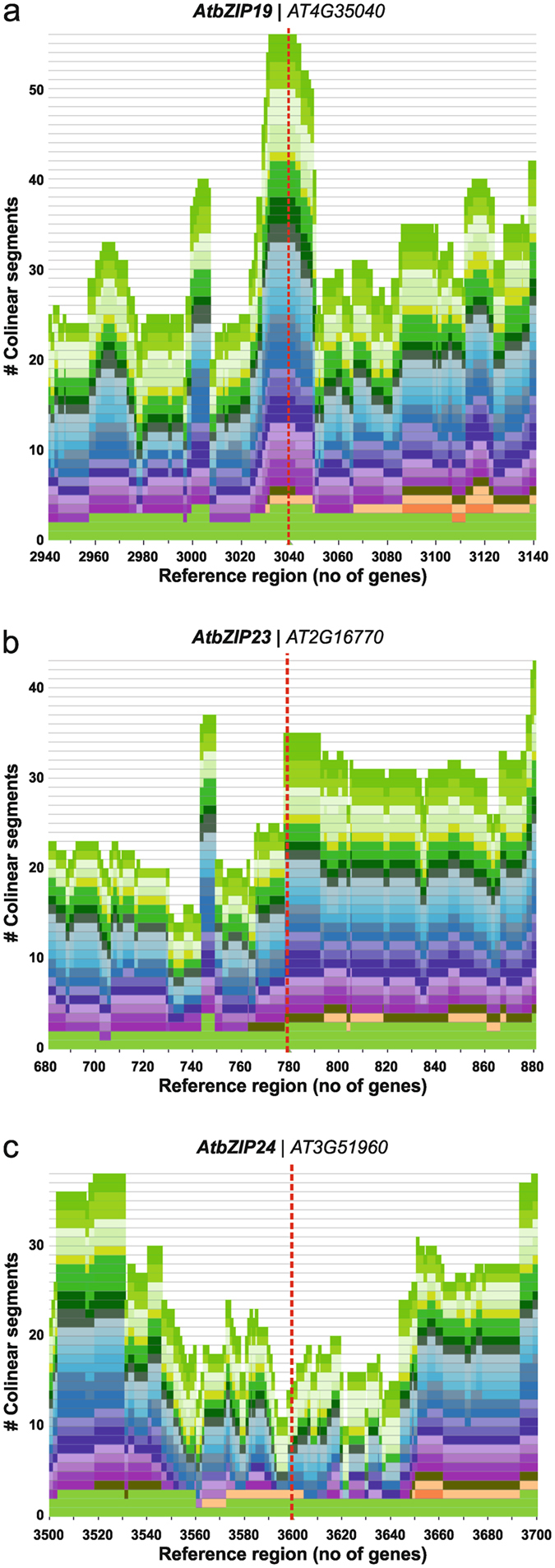
Collinearity analysis for *A. thaliana* F-bZIPs’ genomic regions in comparison to other plants species. Skyline plots were produced with PLAZA, using the AGI codes for *AtbZIP19* (**a**), *AtbZIP23* (**b**) and *AtbZIP24* (**c**) as queries. The red dashed line localizes the position of the F-bZIP gene in the genome segment.

Next, we assessed the extent of genome synteny between orthologous F-*bZIP* genes. The analysis included several Eudicots, Monocots, *A. trichopoda* and *P. patens*, i.e. species available at the Dicots 3.0 Plaza, most of which match our previous phylogenetic analysis. Results for Group 1 showed a high index of collinearity for paralogs of *AtbZIP19*, and medium collinearity for *AtbZIP23* paralogs (Fig. 3a,b). Conversely, a depression in gene collinearity was observed in the 100-gene span surrounding the Group 2 *AtbZIP24* gene, which may be indicative of a lack of selective pressure in this region (Fig. 3c). In addition to between-genome synteny analysis, we specifically looked at within-genome synteny in *A. thaliana*. Group 1 *AtbZIP19* and *AtbZIP23*, but not Group 2 *AtZIP24*, were matched to a large syntenic block containing 102 anchors (Supplementary Fig. S4a), suggesting a more recent duplication event for *AtZIP19* and *−23*. This was also observed across other Brassicaceae species (data not shown), but is lost in remaining Malvids, including the genome of the basal Brassicales species *C. papaya*, which display a single Group 1 gene copy (Fig. 1b). Interestingly, between-genome analysis of this syntenic block indicated that it presented consistently low (<1) ratios of nonsynonymous per synonymous substitutions (Ka/Ks) (Supplementary Fig. S4b), which are indicative of stabilizing selection in Group 1 F-bZIPs.

**Figure 4:**
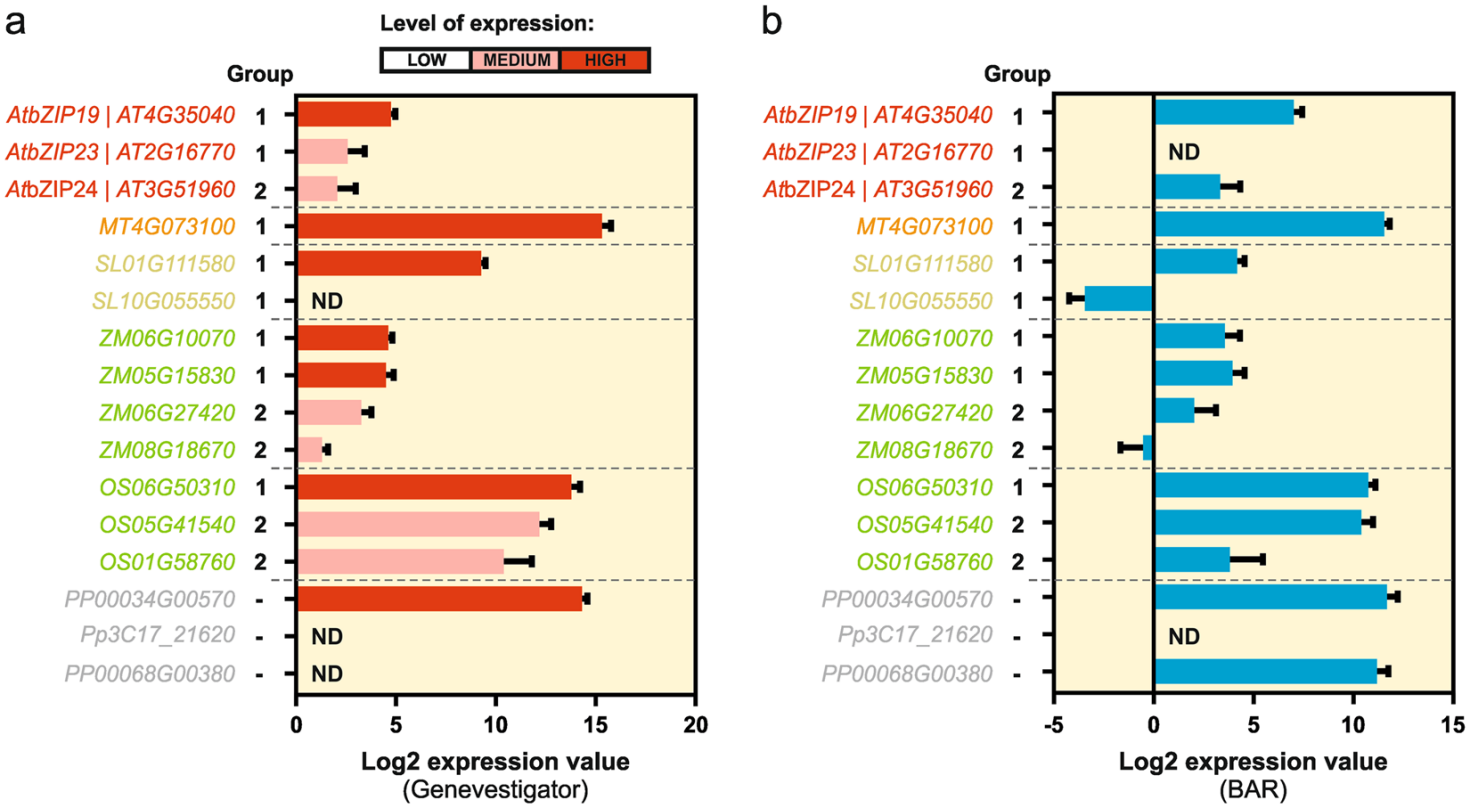
Plant F-bZIP gene expression levels. (**a**) The expression level of each F-bZIP gene along the plant development of each species was retrieved from the Genevestigator database as Log2 of the expression value. The bar on top of the graphic indicates Genevestigator’s classification of expression values as Low, Medium or High, based on quartile expression analysis across all genes. (**b**) The absolute expression values along plant development were downloaded from the BAR database and mean of Log2 calculated. Error bars represent standard deviation (n > 5). ND stands for No Data.

Gene expression data is another valuable resource to decipher the evolutionary trajectory of F-bZIPs. We therefore mined systematic gene expression datasets from six representative species, i.e. *A. thaliana*, *M. truncatula, S. lycopersicum*, *Z. mays*, *O. sativa* and *P. patens*, in order to compare the transcriptional profile of the different F-bZIP groups (Fig. 4a,b). Data indicated that the majority of the *F-bZIP* genes from Groups 1 and 2 are expressed, though with varying expression levels. The analysis portrays average expression across the different developmental stages of each species. It consistently depicts Group 1 genes as having relatively higher expression when compared to Group 2 genes (Fig. 4a,b), suggesting a higher functional significance for Group 1 F-bZIPs, or the existence of different types of regulation, positive or negative control, between the Groups 1 and 2 respectively.

### Transcriptomic profiling of the *A. thaliana bzip19bzip23* mutant in response to contrasting zinc supply

**Figure 5:**
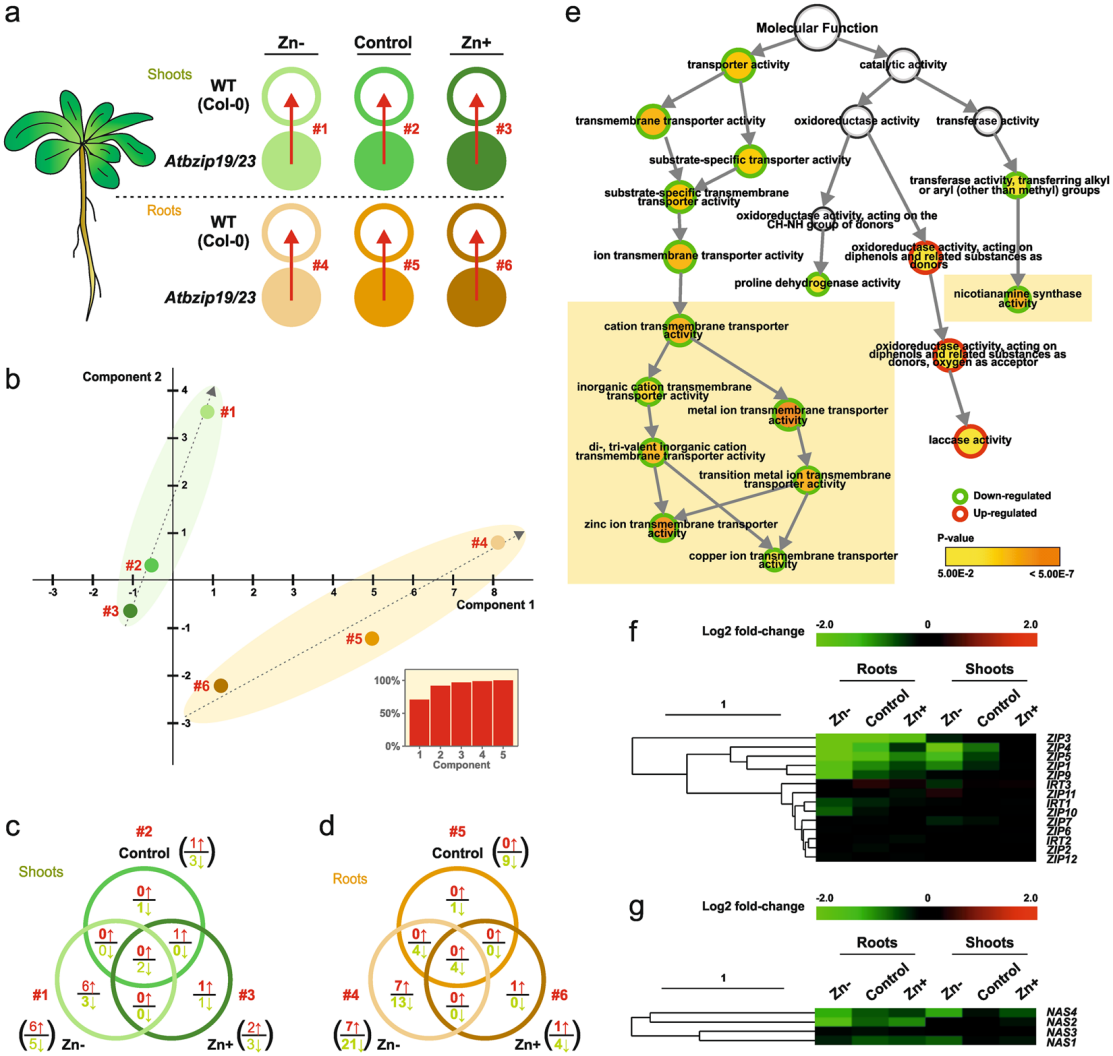
Transcriptomic analysis of the *A. thaliana Atbzip19/23* double mutant in response to contrasting zinc supply. (**a**) Experimental design. Three zinc treatments, Zn- (0.05 µM), control (2 µM), and Zn+ (25 µM) were applied to wild-type (WT) plants (*A. thaliana* Col-0 ecotype) and *Atbzip19/23* double mutant. Tissues from roots and shoots were collected separately^36^. Differentially expressed genes (DEGs) were obtained by comparing the mutant to WT. A number code (#1 to #6) was used to denote all the comparisons. (**b**) Principal component analysis for the DEGs in the 6 comparisons. The inset graph represents accumulative Eigen-values for the components. (**c**) Venn diagram comparison of DEGs in the shoots of *Atbzip19/23*. (**d**) Venn diagram comparison of DEGs in the roots of *Atbzip19/23*. (**e**) Gene Ontology analysis of the DEGs in the *Atbzip19/23* roots. Analysis was performed in BiNGO app of Cytoscape and selecting the *Molecular Function*. The down-regulated and up-regulated categories are highlighted in green and red, respectively. The node colour represents *p*-value associated to each enriched category. (**f**) Clustering analysis of the *A. thaliana ZIP* gene family by expression level in the microarray. The gradient bar represents level of expression from down-regulated (green) to up-regulated (red). (**g**) Clustering analysis of the *A. thaliana NAS* gene family by expression level present in the microarray. The gradient bar represents level of expression from down-regulated (green) to up-regulated (red).

*A. thaliana* bZIP19 and bZIP23, presently annotated as Group 1 F-bZIPs (Fig. 1a), have been shown to function as regulators of the zinc deficiency response^32, 34^. With the aim of obtaining additional biological insight into the AtbZIP19/23 regulatory function, we performed a transcriptomic profiling of root and shoot tissues of the double mutant *Atbzip19-1 Atbzip23-1* (herein as *Atbzip19/23*) and wild-type genotypes exposed to zinc deficiency, sufficiency, and excess (Fig. 5a)^36^. Principal component analysis (PCA) of gene expression variance in raw full expression data highlighted that datasets were mainly resolved between root vs. shoot conditions^36^. Here we conducted PCA analysis of only the differentially expressed genes (DEGs), and again they neatly resolved root vs shoot comparisons, but also the gradient of zinc supply (Fig. 5b). To prevent introduction of background noise, we avoided shoot vs root comparisons in our differential expression analysis, and instead compared wild-type vs mutant under different zinc supply conditions (6 comparisons in total; Fig. 5a). Venn diagrams show that the number of DEGs was relatively small, ranging between 4 and 28 for each comparison (Fig. 5c,d). Most of these genes were down-regulated in the mutant. The highest number of DEGs was observed in the zinc deficiency comparisons, in both shoots and roots, with the majority being down-regulated in roots in the mutant (Fig. 5c,d). These included previously reported putative target genes of AtbZIP19/23, associated with zinc homeostasis, i.e. nicotianamine synthetase (NAS) genes (*NAS2/4*) and members of the ZIP family of metal transporters (*ZIP1/3/4/5/9*)^32^ (Supplementary Table S2). In line with this, identification of the molecular processes statistically associated with DEGs in zinc deficiency in roots clearly included down-regulation of ion transmembrane transporter activity as well as nicotianamine synthase activity (Fig. 5e, Supplementary Table S3). A representation of gene expression values, corresponding to the *ZIP* family of metal transporter genes detected in the present transcript profiling, is shown in a heat map (Fig. 5f). It suggests that the expression of most of the differentially expressed genes decreases with increasing zinc availability. An indication of a similar zinc-dose responsive expression pattern was not observed for *NAS* genes (Fig. 5g).

### *In vivo* reporter assay of *AtZIP4* in *A. thaliana* wild-type and the *Atbzip19/23* mutant

**Figure 6:**
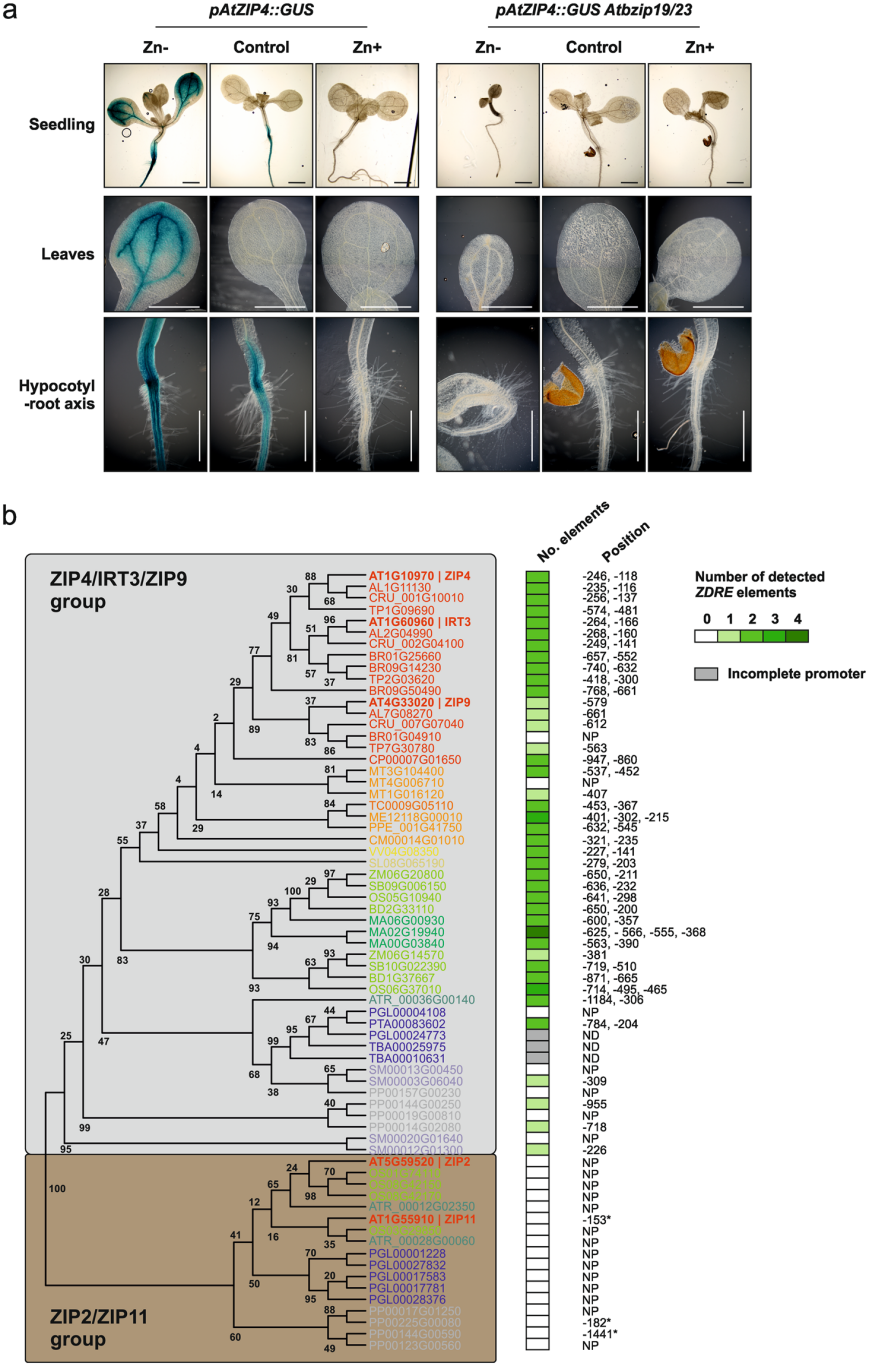
Expression of *AtZIP4* in the *Atbzip19/23* mutant background in response to zinc supply and conservation of the *ZDRE cis*-element in AtZIP4 orthologs. (**a**) *In vivo* reporter assay of *AtZIP4* in *A. thaliana* wild-type and *Atbzip19/23* mutant. Expression profile of *pAtZIP4*::*GUS* lines in wild-type and in *Atbzip19/23* mutant background, in response to zinc supply (Zn- with 0 µM, control with 15 µM, and Zn + with 150 µM). The expression was assessed by submitting 10-day-old seedlings to histochemical B-glucoronidase (GUS) staining. Scale bar indicates 1 mm. (**b**) Conservation assessment of AtbZIP19/23 regulatory mechanism across land plants. Phylogenetic analysis of the *ZIP* gene orthologs of *AtZIP4/9/IRT3* group and *ZIP2/ZIP11* group^37^, with the analysis of the number and position of detected *ZDRE* elements in each gene promoter. Asterisks refer to detected *ZDRE* elements with two mismatches from the consensus sequence. NP stands for None Present and ND stands for No Data.

To obtain additional information on the functional association between AtbZIP19/23 and their putative target genes from the ZIP family of metal transporters in the context of different zinc supply, we studied the expression of *AtZIP4* and the regulatory impact of AtbZIP19/23 loss of function in an *in vivo* reporter assay. An *A. thaliana* line stably transformed with a construct harbouring the *AtZIP4* promoter fused to β-glucuronidase (*GUS*) was introgressed into the *Atbzip19/23* mutant background. The GUS reporter system was used to infer *AtZIP4* expression in seedlings grown in zinc deficiency, sufficiency and excess supply (Fig. 6a). In the transgenic line with wild-type background, we observed a strong GUS staining in roots and shoots of seedlings grown at zinc deficiency, a weaker but visible staining only in the root of seedlings grown at zinc sufficiency, and no visible staining in seedlings grown with excess zinc. In shoots, the expression was visible in the vascular tissue and leaf edges, and in roots there was a strong staining starting from the hypocotyl base. Most significantly, the *Atbzip19/23* mutations completely suppressed expression of *AtZIP4*, irrespective of the zinc supply or tissue involved. This is consistent with the transcriptomic profiling, which showed the *AtZIP4* gene as a DEG in roots and shoots at zinc deficiency, and in roots at zinc sufficiency conditions (Fig. 5c,d; Supplementary Table S2). The previously reported zinc deficiency-hypersensitive phenotype of the *Atbzip19/23* mutant^32^ was also observed, with seedlings showing poor development in comparison with the ones grown at zinc sufficiency and excess supplies (Fig. 6a).

### Assessment of the conservation of the AtbZIP19/23 regulatory mechanism

The previously reported set of putative target genes of AtbZIP19/23, including *AtZIP4*, were shown to contain the *ZDRE cis*-element in their promoter region^32^. In order to evaluate whether there is conservation of this regulatory mechanism across different plant taxa, we performed a survey on the promoter regions of *AtZIP4* and *AtZIP2* gene orthologs, the latter being an unlikely target of AtbZIP19/23^32^. Based on the phylogenetic tree of the annotated 15 *A. thaliana* ZIP family members, the AtZIP4 and AtZIP2 belong to two different groups formed by AtZIP4/9/IRT3 and AtZIP2/11, respectively^37^. Orthologs of the AtZIP4/9/IRT3 group were retrieved from the same set of representative plant species previously used in F-bZIP phylogenetic analysis (Supplementary Fig. S5), whereas orthologs of the AtZIP2/11 group were retrieved from five species representing major taxa (*P. patens*, *P. glauca*, *A. trichopoda*, *O. sativa, A. thaliana*). The resulting sequences were subsequently employed in phylogenetic reconstruction and the promoter regions of the *ZIP* orthologs were analysed for the presence of the 10-bp imperfect palindromic *ZDRE* sequence [RTGTCGACAY]^32^ (Fig. 6b). Interestingly, the majority of the 48 *ZIP4/9/IRT3* orthologs contained *ZDRE* motifs. Within these, only seven genes, from *S. moellendorffii*, *P. patens*, *P. glauca*, *M. truncatula* and *B. rapa* did not contain the *ZDRE*, and another three genes, from *P. glauca* and *T. baccata* had incomplete promoter sequence information. Thus, most of the species had orthologous *ZIP* genes with *ZDRE* motifs in their promoters. Conversely, the promoters of the 15 *ZIP2/11*-orthologous genes did not contain the *ZDRE* motif (Fig. 6b).

The number of observed *ZDRE* motif sequences in the analysed promoters ranged from one to four, but most of them displayed two *ZDRE*. Interestingly, the presence of two elements in the promoter is only observed in seed plants onwards, i.e. *P. patens* and *S. moellendorffii*, which represent older evolutionary lineages, have only one *ZDRE* in their promoters. A total of 76 *ZDRE* motif sequences were found, with up to 1 mismatch allowed. The most common variation to the motif sequence was RTGT/ACGACAY (Supplementary Fig. S6) with 54 elements having no mismatch and 17 elements having 1 mismatch with the latter variation (Supplementary Table S4 and S5).

## Discussion

### F-bZIPs show seed plant divergence into two branches subjected to different selective pressures

We presented a phylogenetic analysis of the plant F subfamily of bZIP TFs, with the aim of shedding light onto their evolutionary history. Our analysis showed that this is a small subfamily of plant bZIPs, which in seed plants diverged into two branches. We did not detect F-bZIP orthologs in the genomes of green algae, which is consistent with the proposal that F-bZIPs emerged in the first terrestrial plant lineage^23^. In seed plants, two branches (termed Group 1 and Group 2) diverged from a monophyletic root containing genes from older evolutionary lineages, i.e. Bryophytes and Pteridophytes. These species (*P. patens* and *S. moellendorffii*) showed F-bZIP duplicates. Considering that duplicates are a major source of evolutionary novelty^16–18^, they may have been relevant in further subfunctionalization among F-bZIPs. The F subfamily comprises, on average, two to three members per species, with the most notorious gene expansions being observed in *M. acuminata* and *B. rapa*, with six members each. This is likely traceable to the recent whole genome duplication and triplication events that have been attributed to *M. acuminata* and *B. rapa*, respectively^38, 39^.

Group 1 and Group 2 genes include the regulators of the zinc deficiency response *A. thaliana* bZIP19 and bZIP23 (Group 1), and the regulator of the salt stress acclimation response bZIP24 (Group 2)^31, 32^. bZIPs are dimeric TFs and predictions of dimerization properties indicated that the three F-bZIPs can form homodimers^8^. Previous works suggests that AtbZIP19 and AtbZIP23 are functionally partially redundant, with indications of AtbZIP19 being the most important player^32, 34^. Our phylogeny suggests that *AtbZIP19* and *AtbZIP23* constitute a lineage-specific pair of paralogs, most likely resulting from the ancestral paleopolyploidization events in the Brassicales lineage, known as the α- and β-duplications (At-α; At-β)^40, 41^. In support, similar *F-bZIP* duplications were observed for other Brassicaceae species, but not for *C. papaya*, which is a Brassicales species that immediately precedes the At-α and At-β WGDs^40^. Moreover, AtbZIP19 and AtbZIP23 are part of a large syntenic block within the *A. thaliana* genome.

Our results suggest that the two distinct groups evolved in seed plants under different selective pressures. Unlike for F-bZIPs in Group 1, not all species display Group 2 F-bZIPs, which suggests parallel gene loss events in the latter. However, our analysis points towards a gene expansion of Group 2. The influence of plant species biology and ecological requirements in the evolutionary fate of gene duplicates has been previously reported^14^. To obtain further insight into the evolution of the two distinct F-bZIP groups, we investigated protein sequence conservation. Previously, the three *A. thaliana* F-bZIPs were defined by the presence of a characteristic His/Cys-rich motif, a signature of this bZIP subfamily, in addition to the conserved bZIP domain of bZIP TFs^24^. Indeed we observed that the His/Cys-rich motif is conserved throughout plant phylogeny, indicating that it might have a differentiating structural and/or regulatory role in F-bZIP activity. A more detailed analysis of the His/Cys-rich motif however, indicates a higher degree of conservation in Group 1 than in Group 2 protein sequences. Although experimental evidence on the role of the characteristic His/Cys-rich motif in F-bZIP activity is still lacking, the identified variations in residue identity and positioning might provide relevant information regarding the potential functional divergence between Group 1 and 2 F-bZIPs, namely the putative neo-functionalization of the Group 2 Brassicaceae AtbZIP24 as a salt stress response regulator. Another differentiating aspect is the presence in Group 1 of a conserved lysine (K) residue, which is interesting given the known relevance of K residues in protein post-translational modification^42^. In line with this, we detected two additional amino acid conserved regions in Group 1 F-bZIP protein sequences, one of which encodes a predicted helical structure, which might contribute to protein conformation. In addition to protein conservation, the analysis of genome synteny provided a strong indication of collinearity for the paralogs of *AtbZIP19*, and to a lesser extent for *AtbZIP23* in inter-specific comparisons. On the contrary, we observed a depression in gene collinearity in the genomic region surrounding *AtbZIP24*, perhaps indicating a lower selective pressure in this region. Finally, F-bZIP gene expression profiles portray Group 1 genes with relatively higher expression than Group 2 genes. These results suggest that following duplication, Group 1 F-bZIPs seem to have conserved their function, whereas Group 2 F-bZIPs became prone to non-/sub- or neofunctionalization as a result of relaxation of selective pressure, or diversifying selection, respectively.

### AtbZIP19/23 are pivotal regulators of the zinc deficiency response and the main regulatory switch of *AtZIP4*

*A. thaliana* Group 1 bZIP19 and bZIP23 regulate the zinc deficiency response, but the underlying regulatory mechanism has not been identified yet. Differential transcriptomics have shown that AtbZIP19/23 control the expression of a small set of genes associated with zinc homeostasis and likely involved in the primary response to zinc deficiency^32^. This gene set comprises *NAS* genes, involved in the synthesis of nicotianamine (NA), a low molecular mass metal chelator with a role in zinc translocation within the plant^43–45^, and *ZIP* metal transporter genes. This family of transporters mediates metal cations influx from outside the cell or from a subcellular compartment into the cytoplasm, and members of this eukaryotic ZIP family are likely involved in plant zinc uptake and transport, though more functional data on plant ZIP proteins are still needed^37, 46–49^. The set of *AtZIP* genes which were shown to have transcript levels upregulated in response to zinc deficiency, but being unresponsive in the *Atbzip19/23* mutant background, and to contain promoter *ZDRE cis*-elements, were suggested as AtbZIP19/23 target genes^32, 48, 50, 51^. Here, we extended transcriptomic profiling of *Atbzip19/23* to zinc sufficiency and excess conditions as well, and not only in roots but also in shoots. Differential expression patterns suggest an almost exclusive role for AtbZIP19/23 in the zinc deficiency response, and highlights a previously unreported role in shoots, including the transcriptional control of *AtZIP4*. Interestingly, the transcriptomics data of *AtZIP* gene expressions indicates that a subset of differentially expressed genes is clearly zinc-concentration responsive. This could be taken to suggest a transcriptional activator function of AtbZIP19/23 being directly or indirectly affected by zinc and inversely proportional to the cellular zinc concentration. The mechanism of regulation of AtbZIP19 and −23 is still unknown and a putative direct or indirect modulation of their activity by zinc (i.e. affecting protein stability, dimerization, subcellular localization, DNA binding activity or transactivation function) needs to be addressed. In particular, the hypothesized role for the His/Cys-rich motif in the direct regulation by zinc of F-bZIP TFs is yet to be determined^35, 52^.

Functional insight into the regulatory mechanism of AtbZIP19/23 and *ZIP* genes was obtained using an *in vivo* reporter assay with the *AtZIP4* promoter. This gene is within the set of AtbZIP19/23 putative *ZIP* targets, with transcript levels strongly up-regulated in response to zinc deficiency, unresponsive in the *Atbzip19/23* background and containing two *ZDRE cis*-elements in the promoter. In addition, ZIP4 mediates zinc uptake in a yeast heterologous system, and the *ZIP4* promoter was successfully used as bait in the yeast-one-hybrid screening leading to AtbZIP19/23 identification^32, 50, 53^. The analysis showed a strong reporter GUS expression in both roots and leaves of zinc deficient seedlings, corroborating previous indications of AtZIP4 involvement in zinc acquisition and distribution in response to zinc starvation^50, 51, 53^. Most significantly, the *in vivo* reporter assay showed that *AtZIP4* is highly regulated by AtbZIP19/23 TFs. These results reinforce *A. thaliana* Group 1 bZIP19/23 as pivotal regulators of the zinc deficiency response, mostly via the activation of *ZIP* genes, and show that, at least for *AtZIP4*, they act as the main regulatory switch.

### Group 1 F-bZIPs function may be conserved in land plants

We also checked the presence of the 10-bp *ZDRE cis*-element in the promoter of orthologous *ZIP* genes from an array of representative plant species. This analysis concerned the orthologs of *AtZIP4* and *AtZIP2*, the latter probably representing a non-target of AtbZIP19/23, without *ZDRE* in its promoter^32, 49^. We found a clear difference in abundance of the *ZDRE* motif in the two groups of *ZIP* orthologs; unlike in the *AtZIP4*-orthologous group, *ZDRE* motifs were not detected in the *AtZIP2*-orthologous group. The search allowed one mismatch but notably most of the detected motifs in the *AtZIP4*-orthologous group contained the complete *ZDRE* sequence motif (ca. 71%), with the remaining ones displaying mostly (ca. 22%) a conserved substitution (i.e. RTGT/ACGACAY). The *ZDRE* represents a unique *cis*-element which does not have the typical ACGT core found in the A-, C- or G-box DNA elements, to which plant bZIPs are known to preferentially bind^24, 54^. A mutated version of the *ZDRE* (i.e. RTGT**A**GACAY) failed to bind AtbZIP19 and AtbZIP23 in an *in vitro* assay^32^, suggesting that it is an essential nucleotide for DNA binding. Further studies are necessary to dissect the role of the nucleotides in the *ZDRE*, as well as understanding whether the sequence-specific DNA binding basic region of bZIP TFs shows specificities in the F-bZIP proteins compared with the other *A. thaliana* bZIPs, which could relate to the AtbZIP19/23 unique ZDRE *cis*-element^4, 24, 32^. Most of the *ZIP* orthologs of the *AtZIP4* group contain two *ZDRE* motifs and this seems to be a pattern emerging in seed plants. In support of the importance of *ZDRE* enrichment, reporter promoter deletion analysis with *AtZIP4*, which contains two *ZDRE*, showed that under zinc deficiency conditions, the presence of the two motifs led to a strong reporter expression, but the presence of the motif closest to the gene start codon alone was not sufficient to induce expression^53^. Similar results were obtained in the yeast-one-hybrid screening using *AtZIP4* promoter fragments as bait^32^. In a preliminary analysis on rice *ZIP* orthologs, we found *ZDRE* motifs in the promoter of Os*ZIP5/7/8/9/10*, reported as being induced by zinc deficiency^55–58^, which supports the conservation of this regulatory mechanism. However, we did not detect the promoter element in the zinc deficiency responsive *OsZIP4* zinc transporter gene (unpublished data), suggesting additional yet unknown regulatory mechanisms. This survey provides a strong indication that the AtbZIP19/23 regulatory mechanism may be conserved across land plants.

### Concluding remarks

The phylogenetic analysis of plant F-bZIPs presented here showed divergence into two groups in the seed plants. Group 1, which include AtbZIP19 and AtbZIP23, appears more conserved, whereas Group 2, which include AtbZIP24, is more prone to gene loss and expansion events. The regulatory mechanism of AtbZIP19/23 is pivotal for the zinc deficiency response in *A. thaliana* and may be conserved across land plants (Embryophytes). There were no F-bZIPs found in available green algae genomes, and in accordance the F-bZIP subfamily was previously predicted to have emerged in the first terrestrial plants, indicating a functional connection to the colonization of the terrestrial environment^23^. It can be envisaged that the evolution of higher organismal and morphological complexity associated with the transition to land^59^, but also the likely new challenges for nutrient acquisition in terrestrial habitats (e.g. variable and fluctuating availability depending on soil properties, biogeochemical and environmental cues)^60, 61^ favoured the emergence of the F-bZIP subfamily TFs from the amplified and diverged green plant ancestors founder genes^23^, with a regulatory function in the response to micronutrient zinc deficiency. Additional studies with other plant model systems (e.g. liverworts) may provide new insights to this hypothesis^62^. Finally, the suggested functional conservation of Group 1 F-bZIPs offers opportunities for translational approaches.

## Methods

### Phylogenetic analysis

Automated gene family annotation resources were used to retrieve the amino acid sequences of F-bZIP ortholgs present in the sequenced genome of the following 24 species: *Arabidopsis thaliana*, *Arabidopsis lyrata*, *Capsella rubella*, *Brassica rapa*, *Thellungiella parvula*, *Carica papaya*, *Theobroma cacao*, *Cucumis melo*, *Prunus persica*, *Medicago truncatula*, *Manihot esculenta*, *Vitis vinifera*, *Solanum lycopersicum*, *Zea mays*, *Sorghum bicolor*, *Oryza sativa ssp. japonica*, *Brachypodium distachyon*, *Musa acuminata*, *Amborella trichopoda*, *Picea glauca*, *Pinus taeda*, *Taxus baccata*, *Selaginella moellendorffii*, *Physcomitrella patens*. Species were selected for being representative of major plant taxa (Bryophyte, Pteridophyte, Gymnosperms, Angiosperms). In addition to the evolutionarily significant *Amborella trichopoda* genome, emphasis was given to Angiosperm taxa containing species of added agronomical significance (Solanaceae, Euphorbiaceae, Fabaceae, Rosaceae, Benincaseae, Malvids including Brassicaceae, and Poales monocots), whose genomes have therefore been sequenced and subsequently characterized in comparative genomic resources such as the Plant Genome Duplication Database, Phytozome and Plaza^63–65^. For support, Fig. 1b depicts an overview of the established phylogenetic relationship of the selected taxa. To search for F-bZIPs across these taxa, web-based platforms Plaza (Dicots 3.0; Dicots 2.5; Monocots 3.0, Gymno 1.0; http://bioinformatics.psb.ugent.be/plaza/)^65, 66^ and Phytozome (v11; https://phytozome.jgi.doe.gov/pz/portal.html)^64^ were scanned with the AtbZIP19 (AT4G35040) query term for gene family assignment on both platforms. Hand curation with sequence alignment analysis was subsequently used to resolve conflicting results. Misannotations were observed for *P. patens*, *S. moellendorffii* and *V. vinifera*. Gene family assignment in these species was estimated following hand curation, supported by BLAST analysis at NCBI (http://www.ncbi.nlm.nih.gov/blast/) against their reference genomes. The complete strategy used to retrieve gene family members is illustrated in Supplementary Fig. S1; gene entries are summarized in Supplementary Table S1.

A similar comparative genomics strategy was used to retrieve the orthologs of the *ZIP* genes *AtZIP4* (AT1G10970) and *AtZIP2* (AT5G59520). Orthologs of the AtZIP4/9/IRT3 group were retrieved from the same set of representative plant species previously used in F-bZIP phylogenetic analysis (Supplementary Fig. S5), whereas orthologs of the AtZIP2/11 group were retrieved from five species representing major taxa (*P. patens*, *P. glauca*, *A. trichopoda*, *O. sativa, A. thaliana*). The search was performed using the web-based platforms Plaza (Dicots 3.0; Dicots 2.5; Monocots 3.0, Gymno 1.0; http://bioinformatics.psb.ugent.be/plaza/)^65, 66^ Phylogenetic analysis was subsequently employed to identify the closest orthologs to the group containing AtZIP4 and the group containing AtZIP2 (Supplementary Table S4 and S5).

Phylogenetic analysis was performed using Cipres (http://www.phylo.org)^67^. First, sequences were aligned with the MUSCLE algorithm^68^, and maximum likelihood inference was then calculated in RaxML v8.2.9 (PROTCAT protein substitution model; LG protein matrix; the 5 D-bZIP gene entries inputted as outgroups; 1000 bootstrap iterations). The final output of the tree was produced using the FigTree software (v1.4.2, http://tree.bio.ed.ac.uk/software/figtree/). The Gblocks^69^ online server (http://phylogeny.lirmm.fr/phylo_cgi/one_task.cgi ? task_type = gblocks), set to minimum stringency, was used to infer on the presence of sequence integrity in the 67 identified F-bZIP genes. The relative abundance of the F-bZIP family in each species was calculated as the average gene family size in the genome across all species. The total number of genes in the genome for each species was determined using the above-mentioned Plaza databases.

### Sequence and synteny analysis, multiple sequence alignment and motif search

The protein multiple sequence alignments were produced using PRofile ALIgNEment (Praline; http://www.ibi.vu.nl/programs/PRALINEwww/) with default settings^70^. Amino acid consistency is calculated by default in Praline^70^, as a linear measure on a scale of 1–10 for consistency in the alignment. To show the similarity between sequences, we used the conservation output where the colour scheme represents the conservation level of each residue in the alignment. The secondary structure prediction of the AtbZIP19 protein was performed using the PSIPRED (v3.3) Protein Sequence Analysis Workbench^71^. To investigate the conserved regions among sequences from different taxon, we used the Multiple Em for Motif Elicitation (MEME, v4.11.2) software, which estimates common motifs and generates their sequence logo representation^72^. Amino acids of interest were subsequently highlighted with different colours.

Identified orthologs of the plant ZIP transporters AtZIP4 and AtZIP2 were subjected to promoter sequence analysis. To that effect, promoter regions were downloaded (in FASTA format) from the Sequence retrieval feature of the Plaza databases (version Dicots 3.0; Dicots 2.5; Monocots 3.0, Gymno 1.0). Promoters consisted of the −2 kb span, or the intergenic region when in the presence of a gene within the −2 kb span. To determine which promoters contained the 10-bp imperfect palindromic *ZDRE* sequence (RTGTCGACAY)^32^, the Motif Alignment & Search Tool (MAST v4.11.2) software^73^ was employed. Only motifs with a position *p*-value inferior to 0.0001 were selected.

Estimation of collinearity between species for a given locus of interest was determined using the Skyline Plot tool in the Plaza Dicots 3.0 database^66^. The window size was adjusted to the genomic span containing 200 genes, and all species available in the database were used in the comparison. Within-genome synteny was calculated with the Locus Search feature at the Plant Genome Duplication Database (http://chibba.agtec.uga.edu/duplication/index/locus), using a 500 kb span^63^. Scores for Ka/Ks ratios between automatically estimated syntenic blocks were retrieved from the Batch Downloads feature at the Plant Genome Duplication Database (http://chibba.agtec.uga.edu/duplication/index/downloads)^63^.

### Transcriptomics and gene expression analysis

A comparative microarray analysis was performed between transcriptome of *A. thaliana* wild-type and *Atbzip19/23* double mutant roots and shoots, with three zinc treatments applied; zinc deficiency, Zn− (0.05 μM), zinc sufficiency, control (2 μM), and zinc excess, Zn+ (25 μM). Experimental design and microarray raw data analysis were previously reported in detail^36^. Raw data files were processed in Bioconductor/R using packages Affy^74^ and Limma^75^. Background correction and quantile normalization were performed using the Robust Multichip Average (RMA) function^76^. Differentially expressed genes (DEGs) were considered as such when displaying adjusted *p*-values < 0.05 following Benjamini-Hochberg correction^77^. Principal component analysis (PCA) was performed in Multi Experiment Viewer (MeV; v4.9.0; http://www.tm4.org/) using DEGs’ expression values. Cross-referencing and Venn visualization of DEGs in different comparisons was performed in Venny v2.1 (http://bioinfogp.cnb.csic.es/tools/venny/). Gene ontology analysis was performed using the BiNGO app of Cytoscape (http://www.cytoscape.org/), selecting for Molecular Function and a *p*-value < 0.05. Only AGI codes corresponding to a unique SpotID were used for this analysis to avoid redundancy and creation of false-positive enrichment. The final output from down-regulated and up-regulated genes were manually combined and the circles were highlighted in green and red, respectively. Cluster analysis of *ZIP* and *NAS* gene family expression in all the selected comparisons were executed in MeV v4.9.0. Clustering was performed using the Hierarchical Clustering tool, with Euclidean Distance as the distance metric. The colour scale limits were set to a +/− 2.0 Log2 range and the midpoint as 0.

*In silico* gene expression analysis during development used expression datasets from Genevestigator^78^ and BAR Arabidopsis, Medicago, Tomato, Maize, Rice and Physcomitrella eFP Browsers (http://bar.utoronto.ca/). The SpotID for each analysed gene is provided in the Supplementary Table S1. For each gene of interest, expression Log2 values were downloaded from Genevestigator using the tool Condition Search – Development. Genevestigator’s classification of expression values as Low, Medium or High is based on quartile expression analysis across all genes for a given species: Low corresponds to genes which expression is in the first quartile, Medium in the interquartiles, and High in the fourth quartile^78^. At the BAR eFP Browsers databases, the SpotID for each gene of interest was submitted and the Developmental Map selected. The values were downloaded as absolute expression values and then Log2 was calculated. The average of the values and standard deviation were calculated in GraphPad Prism software (http://www.graphpad.com/).

### Plant material and growth conditions

Characterization of the *A. thaliana* double T-DNA insertion mutant *Atbzip19/23*, obtained from a cross between homozygous *Atbzip19-1* (SALK_144252) and *Atbzip23-1* (SALK_045200) lines in the ecotype Columbia (Col-0) background, was previously reported^32^. The *Atbzip19/23* double mutant was introgressed into *A. thaliana* Col-0 stably transformed with a construct harbouring the *AtZIP4* gene promoter fused to the reporter gene *GUS* (*pAtZIP4::GUS*)^53^. Homozygous lines were determined by genotyping the F3 generation using the primers depicted in Supplementary Table S6. Synchronized seeds of the *pAtZIP4::GUS* single mutant and *pAtZIP4::GUS Atbzip19/23* triple mutant, were stratified at 4 °C in the dark for three days. Seeds were surface sterilized as previously described^79^ and were sown onto half-strength MS with 1.5% sucrose and adjusted to pH 5.7. The media were prepared without zinc (Zn-, zinc-deficient media), with 15 μM ZnSO_4_ (control, zinc-sufficient media), or with 150 μM ZnSO_4_ (Zn+, zinc-excess media). Plants were grown in a growth chamber with 16 h light/8 h dark cycles (long-days) and a controlled temperature of 22 °C/19 °C (day/night), under cool white light (90 μmol Photon m^−2^ s^−1^).

Histochemical GUS assay of *pAtZIP4::GUS*, in *A. thaliana* wild-type and *bzip19/23* background, was performed as previously described^79^, but omitting the vacuum-infiltration step. Around10 seedlings (10-day-old) per genotype and condition were collected for the assay, and three independent replicates were performed. After overnight incubation, the pigments were removed by repeated incubations in 96% ethanol (v/v), and seedlings were stored in 70% glycerol (v/v). Detailed pictures were made using a Fluorescence Microscope Leica DM 5000B.

## Electronic supplementary material

Supplementary Information
