# Supplementary material for: Phylogenetic analysis of F-bZIP transcription factors indicates conservation of the zinc deficiency response across land plants
Source: Sci Rep. 2017 Jun 19;7:3806. doi: 10.1038/s41598-017-03903-6 (PMC5476651; doi:10.1038/s41598-017-03903-6)
Supplement: Supplementary file 1 — Supplementary Information [file 41598_2017_3903_MOESM1_ESM.pdf]

Phylogenetic analysis of F-bZIP transcription factors indicates conservation of the zinc deficiency response across land plants

Authors

Pedro Humberto Castro, Grmay H. Lilay, Antonio Muñoz-Mérida, Jan K. Schjoerring, Herlânder Azevedo, Ana G. L. Assunção

Supplementary Information

Supplementary Figure S1. Outline of the strategy employed in the annotation of F-bZIPs homologs across a selection of plant taxa.

Supplementary Figure S2. Plant F-bZIP Group 1 protein sequence alignment.

Supplementary Figure S3. Plant F-bZIP Group 2 protein sequence alignment.

Supplementary Figure S4. Synteny analysis in Eudicot F-bZIPs.

Supplementary Figure S5. Phylogenetic tree of ZIP family proteins in various plant species.

Supplementary Figure S6. Sequence logo of the *ZDRE* motif.

Supplementary Table S1. Plant F-bZIPs sequence IDs.

Supplementary Table S2. Differentially expressed genes detected by comparative microarray analysis.

Supplementary Table S3. Gene ontology (GO) enriched categories for Molecular Function of down-regulated genes in *Atbzip19/23* double mutant roots in response to zinc deficiency.

Supplementary Table S4. Annotation of *ZIP* gene orthologs of *A. thaliana ZIP4/9/IRT3*, sequence IDs and presence of *ZDRE cis*-elements in the promoter region.

Supplementary Table S5. Annotation of *ZIP* gene orthologs of *A. thaliana ZIP2/11*, sequence IDs and presence of *ZDRE cis*-elements in the promoter region.

Supplementary Table S6. Primers used for genotyping transgenic *A. thaliana pAtZIP4::GUS* lines in the double mutant *Atbzip19/23* background.

Supplementary Figure S6. Outline of the strategy employed in the annotation of F-bZIPs homologs across a selection of plant taxa. To search for F-bZIP transcription factors homologs in selected plant species (*Arabidopsis thaliana*, *Arabidopsis lyrata*, *Capsella rubella*, *Brassica rapa*, *Thellungiella parvula*, *Carica papaya*, *Theobroma cacao*, *Cucumis melo*, *Prunus persica*, *Medicago truncatula*, *Manihot esculenta*, *Vitis vinifera*, *Solanum lycopersicum*, *Zea mays*, *Sorghum bicolor*, *Oryza sativa ssp. japonica*, *Brachypodium distachyon*, *Musa acuminata*, *Amborella trichopoda*, *Picea glauca*, *Pinus taeda*, *Taxus baccata*, *Selaginella moellendorffii*, *Physcomitrella patens*), gene ID and protein FASTA terms were submitted to Plaza (Dicots 3.0; Dicots 2.5; Monocots 3.0, Gymno 1.0; http://bioinformatics.psb.ugent.be/plaza/) and Phytozome (v11; https://phytozome.jgi.doe.gov/pz/portal.html) databases, respectively. All retrieved protein sequences were aligned using Clustal Omega (http://www.ebi.ac.uk/Tools/msa/clustalo/), and redundant sequences were subsequently removed. Low-confidence sequences (suggesting misannotation by the comparative genomics resources) were observed for *P. patens*, *S. moellendorffii* and *V. vinifera*. In these species, hand-based curation of the gene families was performed: protein FASTA sequences were subjected to BLAST analysis against NCBI annotated genomes, and query results were analysed using Clustal Omega sequence alignment, prior to establishment of bona fide gene family members. The final F-bZIP gene subfamily was summarized in Supplementary Table S1. Sequences were subsequently used in bioinformatic analysis.

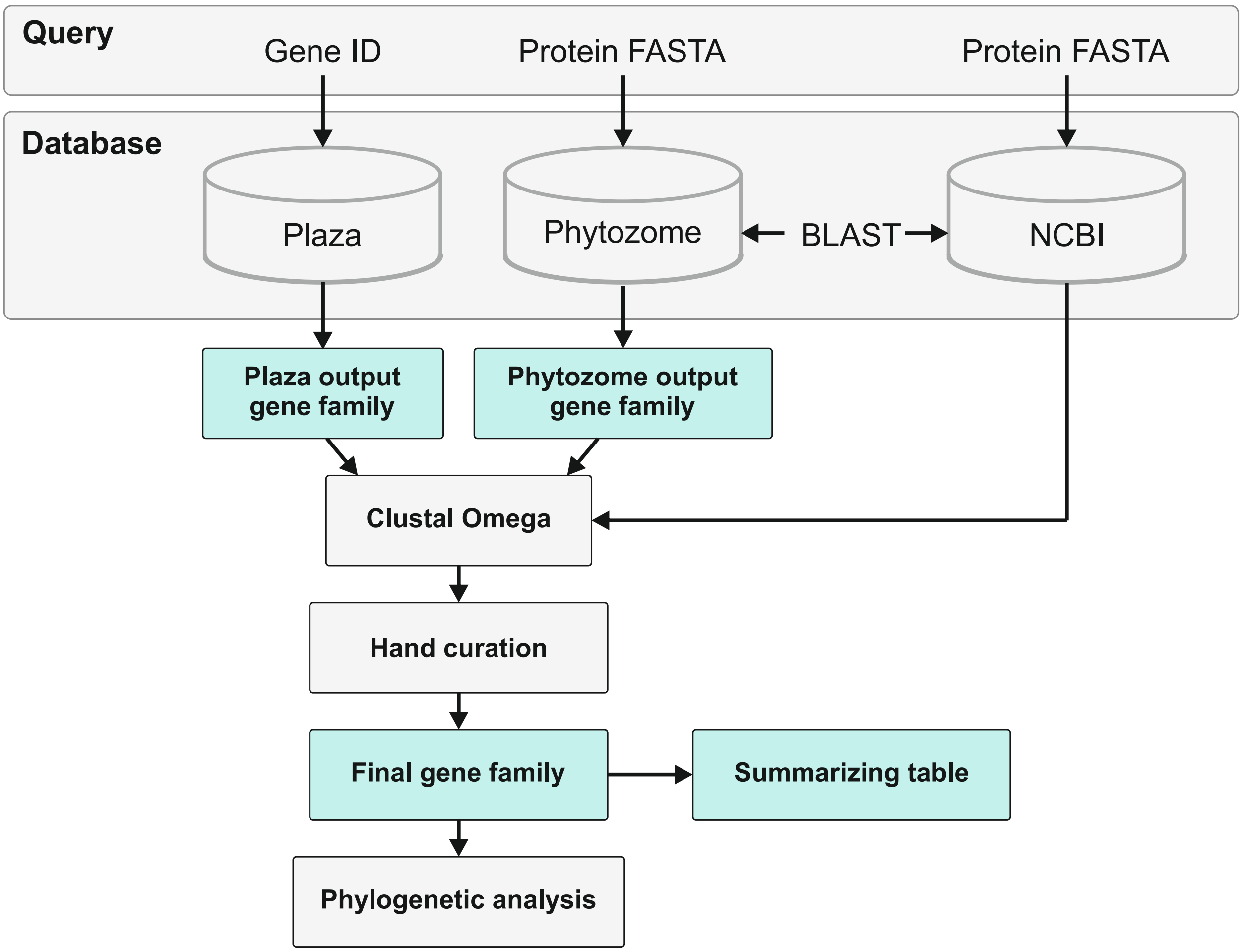

Supplementary Figure S2. Plant F-bZIP Group 1 protein sequence alignment. The protein multiple sequence alignments were produced using PRofile ALIgNEment (Praline; http://www.ibi.vu.nl/programs/PRALINEwww/). Amino acid consistency is classified from 0 (unconserved) to 10 (conserved).

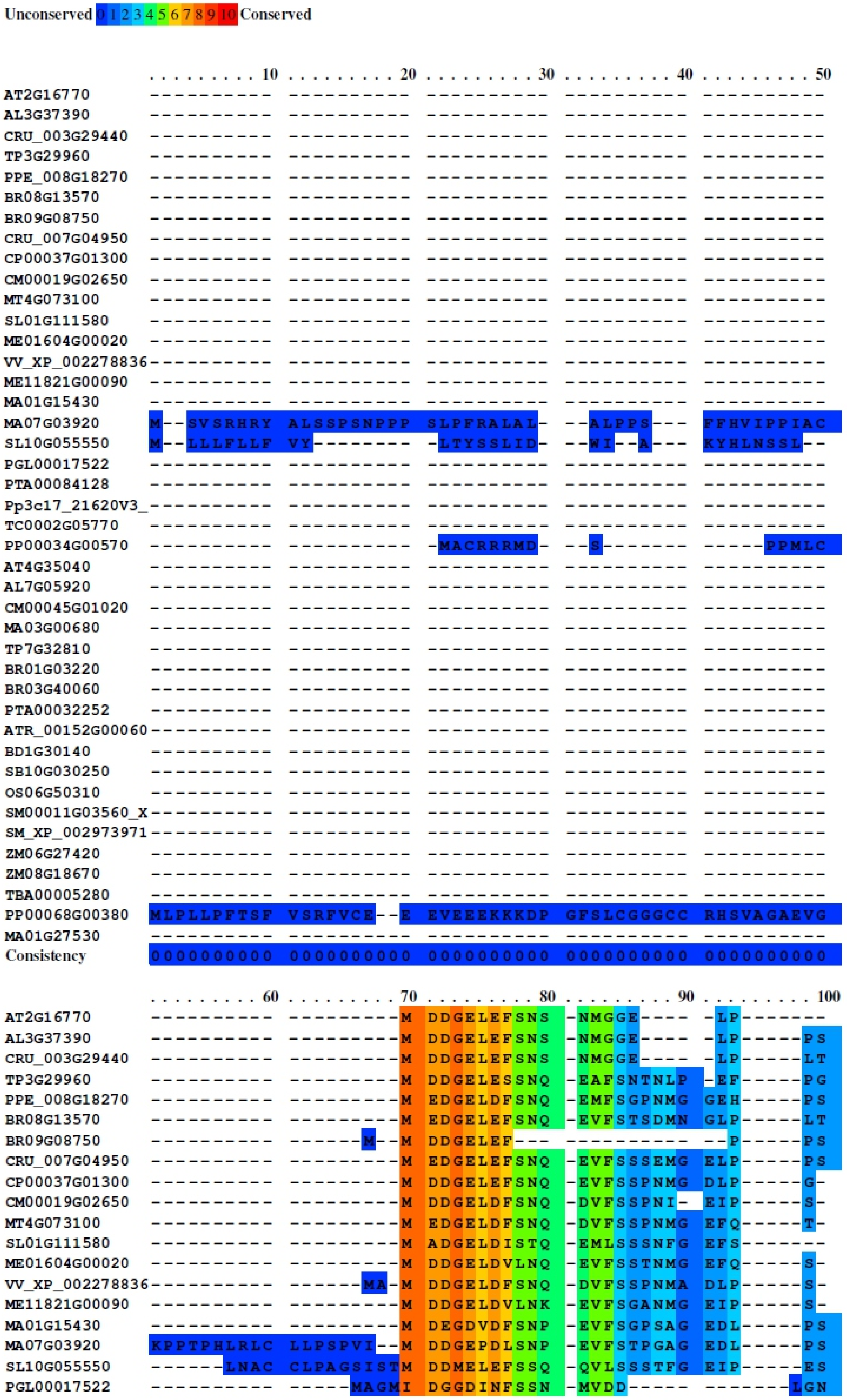

Supplementary Figure S2. (cont.)

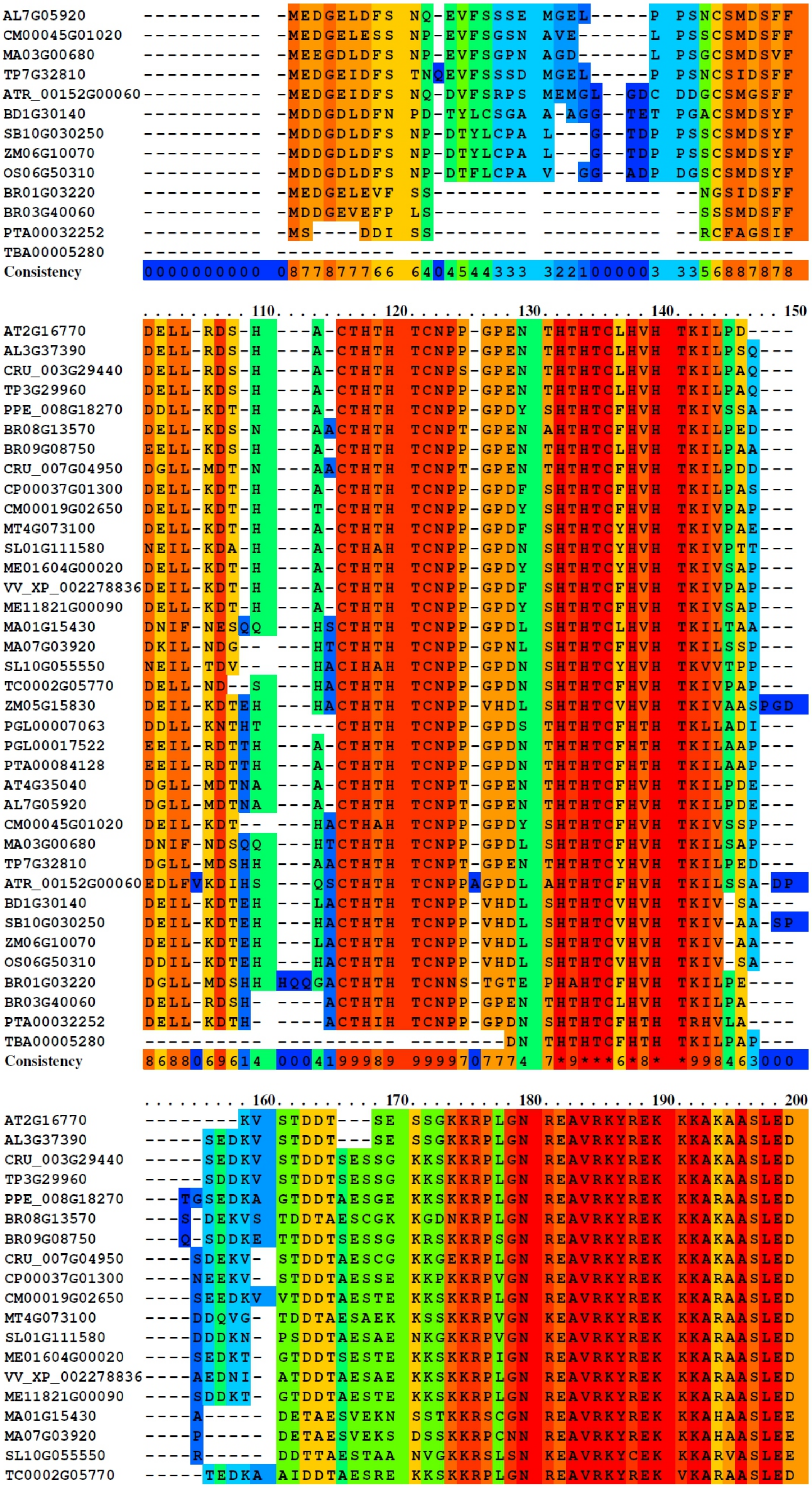

Supplementary Figure S2. (cont.)

```
ZM05G15830        DTADSPSENN TNGSGGNGSN AASKKRPSGN RAAVRKYREK KKAHTASLEE
PGL00007063       ----EEEKSV DNSETHQNGS SKSKKRTSGN REAVRKYREK KKAHTAYLEE
PGL00017522       ----DGEKSA DTAESPQNSS SKPKKRPVGN REAVRKYREK KKARTASLEE
PTA00084128       ----DDEKSA DTAESPQNSS SKPKKRPVGN REAVRKYREK KKARTASLEE
AT4G35040         -----SDEKV STDDTAESCG KKGEKRPLGN REAVRKYREK KKAKAASLED
AL7G05920         -----SDEKV STDDTAESCG KKGEKRPLGN REAVRKYREK KKAKAASLED
CM00045G01020     -----TEEKL STDDTEDSVD KKNKKRPLGN REAVRKYREK KKARAASLED
MA03G00680        -----A---- DETAESAGKS SPTKKRSCGN REAVRKYREK KKAHAASLEE
TP7G32810         -----SDEKV STDDTAESSE KRGEKRPLGN REAVRKYREK KKAKAALLED
ATR_00152G00060   NNDDDAAATT GEEESSSINE QQKKKRPLGN KEAVRKYREK KKARAASLED
BD1G30140         -----SSDGA ESPAENTTSG TSKKRRPSGN RAAVRKYREK KKAHTALLEE
SB10G030250       GDDAAESPSE NNTNTTGNSN AASKKRPSGN RAAVRKYREK KKAHTASLEE
ZM06G10070        ----SPGDAE SPSENNSNGN AASKKRPSGN RAAVRKYREK KKAHTASLEE
OS06G50310        --PSDTPSDA AETAESPTEN NASKKRPSGN RAAVRKYREK KKAHTASLEE
BR01G03220        ----DSDEKV STDDTAESCG KNGEKRPLGN REAVRKYREK KKAKAASLED
BR03G40060        ---------- QADDTSESSG SKSKKTPLGN REAVRKYREK KKAKAASLED
PTA00032252       -----SEGKD SLDESGQNSS SNSRKRPLGN REAVRKYTEK KKAHTASLEG
TBA00005280       --------DEE KSADSTQDSS SKAKKRSGGN REAVRKYREK KKARTASLEE
Consistency       0000134332 5566645555 655899859* 98*****9** 9**67*8**7

                  .........210.........220.........230.........240.........250
AT2G16770         EVMRLKAVNN QLLKRLQGQA ALEAEVTRLK CLLVDIRGRI DGEIGAFPYQ
AL3G37390         EVMRLKAVNN QLLKRLQGQA VLEAEVTRLK CLLVDIRGRI DGEIGAFPYQ
CRU_003G29440     EVMRLRAVNN QLMKRLQGQA ALEAEVTRLK CLLVDIRGRI DGEIGSFPYQ
TP3G29960         EVTRLRAVNN QLLKRLQGQA ALEAEVTRLK CLLVDIRGRI EGEIGAFPYQ
PPE_008G18270     EVVRLRTLNQ QLLKRLQGQA ALEAEIARLK CLLVDIRGRI EGEIGSFPYQ
BR08G13570        EVARLTAVNH QLVKRLQSQG ALEAEVSRLK CLLVDLRGRI DGEIGSFPYQ
BR09G08750        EVLRLRAVNN QLMKRLQGQA ALEAEVTRLK CLLVDIRGRI EGEIGAFPYQ
CRU_007G04950     EVSRLRAVNQ QLVKRLQNQA TLEAEVSRLK CLLVDLRGRI DGEIGSFPYQ
CP00037G01300     EVVKLRALNQ QLLKRLQGQA ALEAEIARLK CLLVDIRGRI EGEIGSFPYQ
CM00019G02650     EVVRLRALNQ HLMKRLQGQA ALEAEIARLK CLLVDIRGRI EGEIGSFPYQ
MT4G073100        EVMKLRALNQ HLMRKLQGQA ALEAEVARLK CLLVDIRGRI EGEIGSFPYQ
SL01G111580       EVVRLRAINQ QLLKRLQGQA VLEAEVARLK CLLVDIRGRI EGEIGSFPYQ
ME01604G00020     EVVRLRTLNQ QLLKRLQGQA ALEAEVARLK CLLVDIRGRI EGEIGSFPYQ
VV_XP_002278836   EVVRLRSLNQ QLLKRLQGQA ALEAEVARLK CLLVDIRGRI EGEIGSFPYQ
ME11821G00090     EVVKLRALNQ QLLKRLQGQA ALEAEVARLK CLLVDIRGRI EGEIGSFPYQ
MA01G15430        EIAQLRLINQ QLVKRLQNQA ALEAEVARLR CLLVDLRGRI EGEIGTFPYQ
MA07G03920        EAAHLRAINQ QLFKRLQNQA ALEAEIARLK CLLVDLRGRI EGEIGPFPYH
SL10G055550       EVVRLRDINQ QLLNRLQGQA VLEAEVSRLK CLLVDIRGRI AGEIGSFPYH
TC0002G05770      EVVRLRALNQ QLLKRLQGQA ALEAEIARLK CLLVDIRGRI EGEIGSFPYQ
ZM05G15830        EVVHLRALNQ QLVKKLQSHA ALEAEVARLR CLLVDIRGRI EGEIGAFPYQ
PGL00007063       EVSHLRALNQ HLIKRLQAQA GLEAEVVRLR CLLADFQHRI DGELGAYPYQ
PGL00017522       EVLHLTTLNQ QLLRRLQGQA ALEAEIARLK CLLADFRGRI DGELGSYPYQ
PTA00084128       QVVQLTTVNQ QLHRRLQGQA ALEAEIARLK CLLADFRGRI DGELGSYPYQ
AT4G35040         EVARLRAVNQ QLVKRLQNQA TLEAEVSRLK CLLVDLRGRI DGEIGSFPYQ
AL7G05920         EVARLRAVNQ QLVKRLQNQA TLEAEVSRLK CLLVDLRGRI DGEIGSFPYQ
CM00045G01020     EVVRLRALNQ QLLKRLQGQA ALEAEVSRLK CLLVDIRGRI EGEIGSFPYQ
MA03G00680        EVAQLRAINQ QLMKRLQSQA ALEAEVARLR CLLVDLRGRI EGEIGSFPYQ
TP7G32810         EVSRLRAVNQ QLLKRLQSQA TLEAENARLK CLLVDLRGRI EGEIGAFPYQ
ATR_00152G00060   EVVHLRAMNH QLMKKLQGQA ALEAEVARLK CLLVDIRGRI EGEIGSFPYT
BD1G30140         EVVHLKALNK ELMKKVQNHA ALEAEVARLR CLLVDIRGRI EGEIGAFPYQ
SB10G030250       EVVHLRALNQ QLMKKLQSHA TLEAEVARLR CLLVDIRGRI EGEIGAFPYQ
ZM06G10070        EVVHLRALNQ QLMKKLQSHA ALEAEAARLR CLLVDIRGRI EGEIGAFPYQ
OS06G50310        EVVHLRALNQ QLMKKLQNHA TLEAEVSRLR CLLVDIRGRI EGEIGAFPYQ
BR01G03220        ECSRLRGLNQ QLVKRLQSQG ALESEVSRLK CLLVDLRGRI DGEIGSFPYQ
BR03G40060        EVIRLRAVNS QLMKRLQGQA ALEAEVTRLK CLLVDIRGRI EGEIGAFPYQ
PTA00032252       EILQLKAQNQ KLMKKIQSQS LLEAEITRLR CLLGDLRSRI DGELTSYLHQ
TBA00005280       EVLHLTALNQ QLMRRLQGQG VLEAEIARLK CLLADFRGRI DGELGSYPYQ
Consistency       9966*877*7 8*7989*688 7**9*87**8 ***8*899** 7**9979999

                  .........260.........270.........280.........290.........300
AT2G16770         KPA------- -----VTNV- ---PYSYMMH PC-NMQCD-V DNLYCLQ---
AL3G37390         KPA------- -----VTNV- ---PYSYMMH PC-NMQCD-V DNLYCLQ---
CRU_003G29440     KPA------- -----AANV- ---PYSYMMH PC-NMQCD-V DNMYCLQ---
TP3G29960         KPA------- -----ATNS- ---PFSFTMH PC-NMPCD-V DSLYCLQ---
PPE_008G18270     KSVNPNL--- ----PNPNI- ---PSAYVMN PC-NLQRD-- DQLYCLHQ-G
BR08G13570        KP-------- -----SVPS- ---FSHMMMN PC-NVEC--G DEVYCLQ---
BR09G08750        KP-------- --------Q- ---PFSYRMQ PC-NMPCD-V DDLYCLQ---
CRU_007G04950     KPNIPS---- ---------- ----FSHMMN PC-NVQCD-- DEVYCPQN-G
CP00037G01300     KPANVNL--- ----PAANM- ---PGAYVMN PC-NMQCD-- DQVYCLHS-G
CM00019G02650     KAVNPNL--- ----SNPSM- ---PGAYVMN PC-NMQCE-- DQVYCLHPGV
MT4G073100        KPANVNP--- ----AMN-M- ---PGSHVMN PC-NVQCD-- DRAYCLRP--
SL01G111580       KPMKSGNTY- -QHIVNPNF- ---PGAYVVN SC-NLQCD-- DQVYCLHP-G
ME01604G00020     KSAKDVN--- ---LANPNV- ---AGAYVMN PC-DVQCN-- CRASCLHP-G
VV_XP_002278836   KSAKSGDGY- -PNMVNQSL- ---SGAFVMN PC-NLQCD-- DQVYCLHP-G
```

Supplementary Figure S2. (cont.)

```
ME11821G00090   KSANELN--- ---FANPNL- ---ASAYVMN PC-NVQCN-- SQAYCLHP-G
MA01G15430      KPVKGSGDIG SNAFQANLL- ---GDAQVLN SC-GFRCD-- DQVSCLHPGM
MA07G03920      KPVKGSGDFV SNVTQGNML- ---AGAQVLN PC-NFHCD-- DQVNWFYPGM
SL10G055550     KPME------ -GADVYQNL- ---RGTYVMN PC-NLQCD-- DQVYCLQP--
TC0002G05770    KSTTNVNMMN L--------- ---PGAYVMN PC-NVQCN-- DQMYCLHP-G
ZM05G15830      RPPPAAKNVD LVSGVDQGGF LAGAAAQVTS SC-DFRCN-- DQMYC-SP-G
PGL00007063     KHV---RTDE QDGTLQLL-- ---PGGYVLN SF-HVPCD-- ADVPCLHP--
PGL00017522     KSIRMDKTCN DAPFRQPM-- ---PGGYVLD PC-NIWCN-- ADAACHDP--
PTA00084128     RSIRMDKTCN DAPFRQPM-- ---PGGYVLD PC-NIWCN-- ADAACRDP--
AT4G35040       KPMAANIP-- ---------- ---SFSHMMN PC-NVQCD-- DEVYCPQN-V
AL7G05920       KPMAANIP-- ---------- ---SFSHMMN PC-NVQCD-- DEVYCPQN-V
CM00045G01020   KPANSNPP-- --NQNVSG-- -----SYMIN PC-NVECN-- DQAYCLRP-G
MA03G00680      RTVKGSGD-- --FVSNVSQA NMLGDAEVLN SC-GFRCD-- DQVSCLYPGM
TP7G32810       KPLPANIP-- ---------- ---SFSHMMN PC-NVHCE-- DETYCLQDGF
ATR_00152G00060 NKPPPSSSVL -QHQQP-LTG GGFALNSCNL EQ-CDNEQ-- LQCFRPSD-K
BD1G30140       RPVKNVDLVS -GGVD----- LLGGGSQVMN SC-DFRCN-- DQLYCNPG-M
SB10G030250     RPPVKNVDLV -SSVDQGSFL GGAGTAQVTN AC-DFRCK-- DQMYCN----
ZM06G10070      RPPAVKNVDL -LSSVDQESL LGSAAAQVAN SC-DFRCN-- HQMYCN----
OS06G50310      RPVKNIDLVS -SVDQG---- SYLGGAQVMN SC-DFRCA-- DQMYCSPG-M
BR01G03220      K--------- ------PNI- --PSFSHLVN PC-NVQC--E DEVYCLGD--
BR03G40060      K--------- ------RVV- --P-NSYTMQ PCNNVPCGVD NSLYCFQD--
PTA00032252     QQPRTDKHGD SSLQIMPGR- --CALSSCNV NLNLVNLSCN ADLPCPHT--
TBA00005280     KSTIRTDK-- ---ATDGQFL QPLPGGYVIN SC-NVRCN-- ADVTCPDPTL
Consistency     8532121100 0001122220 0004365666 6806658500 6666944301

                .........310.........320.........330.........340.........350
AT2G16770       -NGNNGEGAS MNEQGL--NG CEFD-QL-EC LANQNLA--- GKEIPVC--S
AL3G37390       -NGNNGEGGS MNDQGL--NG CEFD-QL-EC LANPNLA--- GKEIPVC--S
CRU_003G29440   -NGNNGEGTL MNEQGL--NG CEFD-QL-EC LANQNLA--- GKEIPLC--S
TP3G29960       -NGNVGEGTS MNQQGL--NG CEFD-QL-QC LGNQNLA--- GKEIPVC--S
PPE_008G18270   ADGKCGDGAV MNGQGF--SG CDFE-NL-QC LVNQDGG--- YKELSGC--G
BR08G13570      -DG-FGGSSQ GGVSIN--DG CGFD-QLQQC VANHNLV--- G-----N--G
BR09G08750      -NGNSGEGIL MNGQGL--NG CEFD-QL-QC LGDQNLA--- G-----C--S
CRU_007G04950   FGGNTQEVAS INDQGL--SG CDFD-QL-QC MANQNLG--- GNA-------
CP00037G01300   VDGRSGEVVS LNGQGV--SG CEFE-HL-QC LAGNNSG--- IKELPAC--G
CM00019G02650   DGSRSSEGAV INGQSF--GA CEFE-NL-QC LANHDSG--- SKELPGC--G
MT4G073100      DGKIAAEVAS PNEEGF--DG CEFE-SL-QC MGGQNLG--- LKDLRGC--G
SL01G111580     AEGKNSDGTV LNGQGF--NN CEFE-TL-QC LGNQTSG--- LEEVPGC--V
ME01604G00020   MDGKNGEGMA LNGQGF--SS CEFD-NL-QC LLNQNSG--- MKELAGCELE
VV_XP_002278836 VEAKNGEAAG LNGQGF--NG CDFE-NI-PC VGNPSAA--- LKELPGC---
ME11821G00090   ADGKSGDGIA LNGQGL--TG CEFD-NL-QC LANQNSG--- MKELAGCGIG
MA01G15430      QGKNIGENGA FNGQGV--RH CEIG-NI-QC MGSSTSG--- SKNFFGC--G
MA07G03920      HGKDVGETGV FDDQGL--GV CEIG-NM-QC MGSSTSG--- SQDFASC--K
SL10G055550     ------NSEE SNGQGL--NS CGFE-TL-QC SSSQGL---- -KEVPGC--A
TC0002G05770    ADGKTGEVAE LNGQGF--NV CEFD-NL-PC LANQNSG--- EKELSTYG--
ZM05G15830      MQ----GAVS GQVLGQ--GA CDVS-SI-QC VGSTRSA--- STKLPVCG-G
PGL00007063     -SITPQRNGG MLSE-D--GS TTKWKEGCNL VAGNCQA--- LK--------
PGL00017522     -TLASNSEGG VQHERD--SG ARWN-GECGQ ISGHCQG--- LK--------
PTA00084128     -TLASNSEGG VQHE-H--DS AARWNGDCGQ IAGHCQG--- LK--------
AT4G35040       FGVNSQEGAS INDQGL--SG CDFD-QL-QC MANQNLN--- GNG-------
AL7G05920       FGVNSQEGAS INDQGL--SG CDFD-QL-QC MANQNLG--- GNG-------
CM00045G01020   DDGKSGESTL LNGQSF--SA CDFE-NL-QC LANQNTG--- AKEPPDC--G
MA03G00680      QGRNVGENGA FNGQGV--QA CEIG-NI-QC MGSSTSG--- YKDCFGC--G
TP7G32810       GGNNSQEGAS INDQGL--GG CEFD-QL-QC MANHNLG--- GHA-------
ATR_00152G00060 SVQEDVPLHN NGGLSQGFGV CDVP-AV-QC GNPLIVGS-- SKEVGGC--A
BD1G30140       QMRTVGDDGA MNGQAFGQGT GDFV-NV-QC LGSAKSG--- STISPGC--G
SB10G030250     ----PGMQGA ISGQVLGQGA CDVA-SI-QC IGSNKSG--- STKLPVC--G
ZM06G10070      ----PGMQGA ISGQVLGQGA CDVA-SM-QC IGSTKSG--- STKLPVC--G
OS06G50310      QVRTMGEDGA VSGQVLGQGA CDIA-SI-QC QGA-KSG--- SAKLPVC--G
BR01G03220      --GFGVG--- -NNRQE--GG GDFD-QL-QC MTD------- --QN------
BR03G40060      --GDGGEGAL MNGQGM--NG CEFD-QL-QC LGGDQDLA-- GCSN------
PTA00032252     --DFQNE--- CYD-AK--NK CQSL-QV-TV HASKGVSV-- CSDC------
TBA00005280     TSEGNGDMQN EHGMCW--NG GCGLMPGPNC QGLKDDVGVT SSGLSACSEG
Consistency     1333356344 4657540055 7664056078 6654445000 3543224002

                .........360.........370.........380.........390.........400
AT2G16770       NGIGTFTVNG SGVNKRK--G EPRA---AKA V--------- ----------
AL3G37390       NGIGTFTVNG SGANKRK--G GPRA---AKA V--------- ----------
CRU_003G29440   NGVGTFTVNG SSTNKRK--G GHRA---AKA V--------- ----------
TP3G29960       NGNGTFSVNA SGANKRK--G GNHA---AKA V--------- ----------
PPE_008G18270   LGNGVSNGNS SATNKRK--G GARA---AAA N--------- ----------
BR08G13570      NGSFNSSANA SASNKRK--G GNKA---A-- ---------- ----------
BR09G08750      NGNGTFDVDA SGANKSK--G ETLT---P-- ---------- ----------
CRU_007G04950   -NGSFNNSNA SVSNKRK--G GHRA---SRA V--------- ----------
CP00037G01300   AGNAATNVS- SGANKRK--G ETHS------ ---------- ----------
```

Supplementary Figure S2. (cont.)

```
CM00019G02650      VGNAVSTDIS SGATKKK--G GSRK---ETW T--------- ----------
MT4G073100         GGPALSNVNS QASSKRK--G GSRA---AKA G--------- ----------
SL01G111580        VGNSTPTDNT SGRSKRK--G GART---TS- ---------- ----------
ME01604G00020      NVVTIVNGNS SSTKKRK--G GT-------- ---------- ----------
VV_XP_002278836    GVGNGITANS SIPNKRR--R GSGS---NS- ---------- ----------
ME11821G00090      NLVTNANGNS SSTNKRK--G GTRG---ATG G--------- ----------
MA01G15430         RGPARQVDCS SNTTKIE--G AHAAE-DL-- ---------- ----------
MA07G03920         SKTAKPVDCS SNATRLE--G ARAPE-DS-- ---------- ----------
SL10G055550        LGFGTPTANA SRGNKKKRTG GKRAH-NGKP VNC------- ----------
TC0002G05770       VGSAGSNGNS SGTKRRK--D FREGGISLMF QYCEPFVGEL QVSL--PAVV
ZM05G15830         MDAVPAACLP NVQNKTP--G SVYG--PFLF RDCWDFPFCL EGTLYKPLAF
PGL00007063        VN-EAGSCGV VACPE----D TMAN---VVP AAVPSENLIS HCSM---TK-
PGL00017522        GDMAVTSSGL SACSE----G TATK---TVP AAVASSGKER KGAF---GV-
PTA00084128        GDMAVTSSGL SGCSE----G TATK---TVP AAVASSGKER KGAF---GV-
AT4G35040          -NGSFSNVNT SVSNKRK--G GHRA---SRA V--------- ----------
AL7G05920          -NGSFNNGNT SVSNKRK--G GHRA---SRA V--------- ----------
CM00045G01020      LGNTIANVNC AELNPKK--G GG-------- ----VCKATK NG--------
MA03G00680         NGAA-TPVDC SSNAKKI--G GA-------- ----HATKED L---------
TP7G32810          -NGSLSNANA SISNKRK--G GHRA---PRA V--------- ----------
ATR_00152G00060    YGNPLPAVVP STSRRRR-TR GTDG------ ---------- ----------
BD1G30140          GMSNMPFGCL PNAKK----- ---------- ---------- ----------
SB10G030250        GMDTVPAVCL PNVENK---- ---------- ---------- ----------
ZM06G10070         GLDTLPAVCL PNVEKK---- ---------- ---------- ----------
OS06G50310         AMGTMPVGCM PNSEKK---- ---------- ---------- ----------
BR01G03220         LNGSFNSANV SASNKRK--G GV-------- ---------- ----------
BR03G40060         GNGTFGVNAS GVGNKRK--R GT-------- ----FAAKAV ----------
PTA00032252        SLTSMGPIDI SPGEQRK--T GV-------G MPQCLSDRSV SG--------
TBA00005280        AVTNTIPAIM TSGGKQK--K GTFA---A-- ---------- ----------
Consistency        3435334444 7445855005 5323000211 1000000000 0000000000

                   .........410.........420.......
AT2G16770          ---------- ---------- -------
AL3G37390          ---------- ---------- -------
CRU_003G29440      ---------- ---------- -------
TP3G29960          ---------- ---------- -------
PPE_008G18270      ---------- ---------- -------
BR08G13570         ---------- ---------- -------
BR09G08750         ---------- ---------- -------
CRU_007G04950      ---------- ---------- -------
CP00037G01300      ---------- ---------- -------
CM00019G02650      ---------- ---------- -------
MT4G073100         ---------- ---------- -------
SL01G111580        ---------- ---------- -------
ME01604G00020      ---------- ---------- -------
VV_XP_002278836    ---------- ---------- -------
ME11821G00090      ---------- ---------- -------
MA01G15430         ---------- ---------- -------
MA07G03920         ---------- ---------- -------
SL10G055550        ---------- ---------- -------
TC0002G05770       ACDSL-G--S YK-LACIAFS NSYSGFA
ZM05G15830         LCRAESGSNC YVFLRCSPSP MKCSN--
PGL00007063        ---------- ---------- -------
PGL00017522        ---------- ---------- -------
PTA00084128        ---------- ---------- -------
AT4G35040          ---------- ---------- -------
AL7G05920          ---------- ---------- -------
CM00045G01020      KC-------- ---------- -------
MA03G00680         ---------- ---------- -------
TP7G32810          ---------- ---------- -------
ATR_00152G00060    ---------- ---------- -------
BD1G30140          ---------- ---------- -------
SB10G030250        ---------- ---------- -------
ZM06G10070         ---------- ---------- -------
OS06G50310         ---------- ---------- -------
BR01G03220         ---------- ---------- -------
BR03G40060         ---------- ---------- -------
PTA00032252        -C-------- ---------- -------
TBA00005280        ---------- ---------- -------
Consistency        0000000000 0000000000 0000000
```

Supplementary Figure S3. Plant F-bZIP Group 2 protein sequence alignment. The protein multiple sequence alignments were produced using PRofile ALIgNEment (Praline; http://www.ibi.vu.nl/programs/PRALINEwww/). Amino acid consistency is classified from 0 (unconserved) to 10 (conserved).

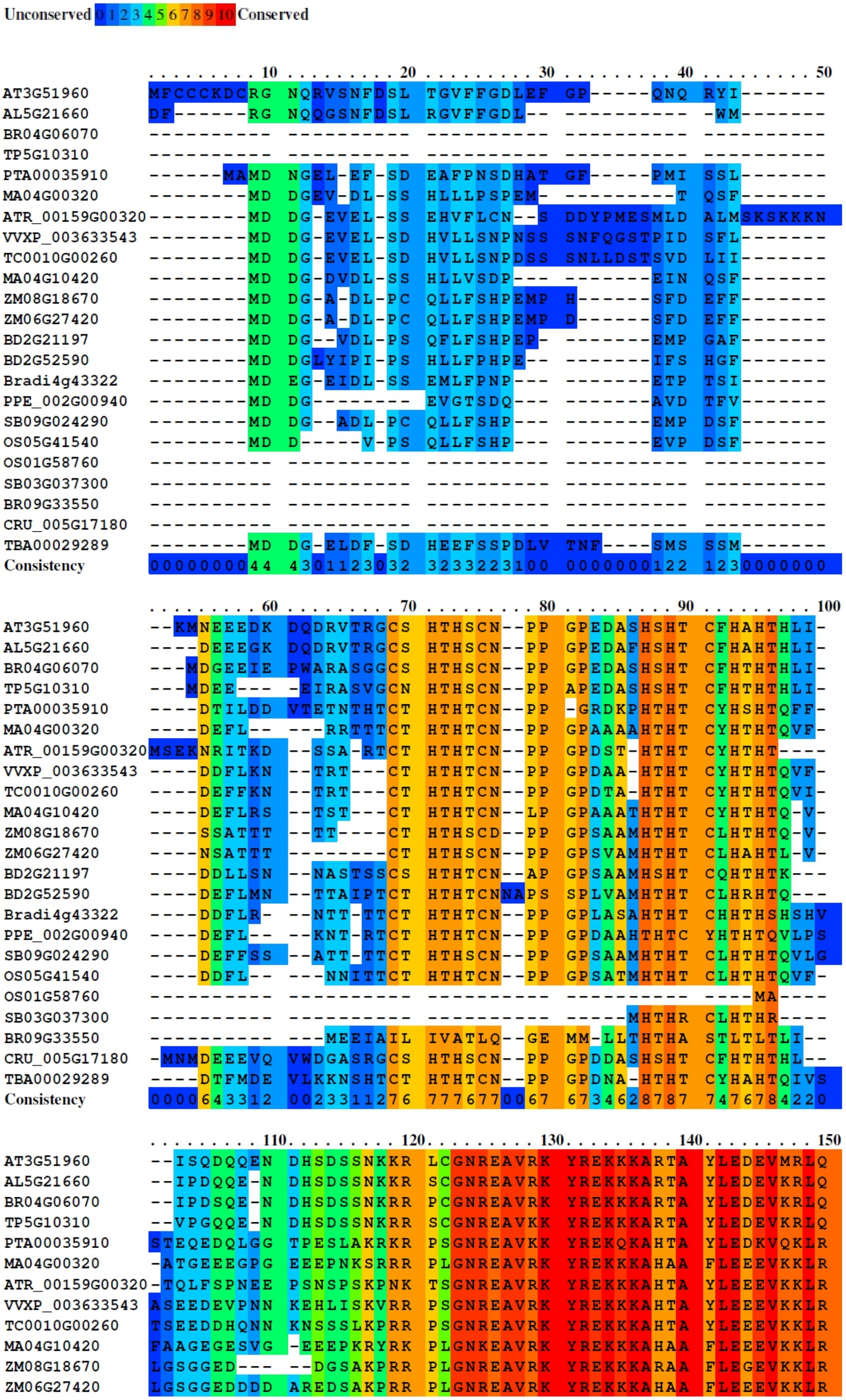

Supplementary Figure S3. (cont.)

```
BD2G21197         -VFATGSEDD DGNPAAKTRR PLGNREAVRK YREKKKARAA FLEEEVRKLR
BD2G52590         -VLASAE--- QEPRNPRVIK PLGNREAVRK YREKKKAHAA FLEEEVRSLR
Bradi4g43322      FSTDDDSCGS DKVVPKKTRE PLGNRVAVRK YRQKKKAHTA HLEEEVRRLR
PPE_002G00940     EDDDGAKNKE HSVPRPRRRR PSRNREAVRK YREKKKAHTA YLEEEVKKLR
SB09G024290       SGGEDDDDAR EEEDSAKPRK PLGNREAVRK YREKKKAHAA FLEEEVKKLR
OS05G41540        --ASGSGEDD IKEDLTKTRR PLGNREAVRK YREKKKAHAA FLEEEVKKLR
OS01G58760        -----SGENN VEEELRKTRK PLGNREAVRK YREKKKAHAA FLEEEVKKLR
SB03G037300       -----QFIAS SSEELRTPRK PLGNREAVRK YREKKKAHAA FLEEEVKKLR
BR09G33550        --IPDPQ--- -ENDNSDISG HVGTERQRGK YREKKKARTA YLEDEVKRLQ
CRU_005G17180     --IIPDQQEN DHSDSSNKKR SCGNREAVRK YREKKKARTA YLEDEVKRLQ
TBA00029289       SEEEKGLVEK SEQFTETKKR PLGNREAVRK YREKKKAHTA YLEEEVQKLR
Consistency       0133344324 3454456477 659998998* **9*9**77* 7**79*88*8

                  .........160.......170.........180.........190.........200
AT3G51960         SLNEQFLRKL QSQEMVETEL IRLRALLVEM QGKIEVELCS -FSFQKQCNG
AL5G21660         SLNEQMLRKL QSQEMMESEL IRLRTLVVEM QGKIDVELCG -FSFQKQCNG
BR04G06070        SMNEFLLRKL QSQAIVEAEI IRLRTLLAEM QKTIDDELGG -FSFQKQCNG
TP5G10310         FLNEHLIRKL QSQATVETEV IRLRTLLVGI QGNIDGELGG -FSFQKQSND
PTA00035910       SLNQQLWRKV QDQVALEAEA ERLRSILFEF KGKIDSELGS -VPYQKPCSG
MA04G00320        LLNQQLLRRL QGQAALEAEV VRLRDLLADL RGKIDAEVGG -GFPLLLKKC
ATR_00159G00320   ALNQQLLRTL QGQAMLEAEV VRLRTILMDL RGKIDGELGV -SPFQRASTI
VVXP_003633543    LLNQQLVKKL QGQAVLEAEV LRLRSLLLDL RGKIDNELGV -FPFQKQCNT
TC0010G00260      LLNQQLVRKL QGQTILEAEV LRLRSLLVDL RAKIDGGLGV -FPFQKQCNN
MA04G10420        LVNQQLLRRL QGQAALEAEV IRLRNLLVDF RGKIDAELGG -FPFQNQCRP
ZM08G18670        AANQQLVRRL QDHAALEAEV ARLRGLLLDV RGRIDAEVGV -SPFQRPCSA
ZM06G27420        AANQQLQRRL QGHAALEAEV ARLRGLLLDI RGKIDAEVGG VLPFQKPCSV
BD2G21197         AANQQLLRRL QGHGALEAEV VRLRSLLPDV RAKIDAEVAV -PVAVTPFQK
BD2G52590         AANQQLLRRL QGHAALEAEV VRLTSLLFDV RAKIDAEI-- ---GDLPLQQ
Bradi4g43322      AINQQLVKRL QGQAALKAEV VRLRTLLVDV RAKIDGALGS -YPFQTQCGV
PPE_002G00940     LLNQQLVRKI QGQAILETEF LRLKSVFLDL RGKIDHELGA -FPFQNQYLP
SB09G024290       AANQQLLRRL QGHAALEAEV ARLRSLLLDV RGKIDAEVGV -FPFQRPCSV
OS05G41540        AANQQLLKRL QGHAALEAEV IRLRSILLDV RGKIDMEIGT -FPYQKPCSV
OS01G58760        TTNQQLLRRL QGHISLEAEV VRLRALLFDI RGKIDAEIGT F-PFQKQCSF
SB03G037300       ATNQHLLRRL QRHAALEAEV VRLRGLLFDV QGKIDAEIVG AFPFQKHRSF
BR09G33550        SLNEYLLRKL QSQRMVETEI IKLRALLVEM QVKIDDELGV -FSFQKQCNG
CRU_005G17180     TLNEHLHRKL QSQATVETEV IRLRTLLVEM QGKIDGELGG -FSNQKHCFG
TBA00029289       LLNQQLLRKL QVQA------ ---------- ---------- ----------
Consistency       56*8796979 *576578797 6898588576 7679847753 0446754542

                  .........210.........220.........230.........240.........250
AT3G51960         SGFVFKEDGC NLA------- ---------- ---TSNMMCE AARVECEEGQ
AL5G21660         SGFVYKEDGC SVA------- ---------- ---TSNMMCE AARVECEEGQ
BR04G06070        SGFVFKEGQI DHNTISILCN NTD-----GC NVATRNMICE VARVECEEGK
TP5G10310         SGFVFKEGRM DRHTISVPCN ---------- --HTMNVMCE VPRAECEESK
PTA00035910       NVKG------ ---------- ---------- ------GDCS VQPLNGDHNL
MA04G00320        SPAGVHCDS- ---------- ---------- ---NGQCVEV IDWEGSCVPV
ATR_00159G00320   CGSGFKEGNC SLGSIHGVGE VGLNLVGCGD DVPPCLHPQG KVMENCEPPI
VVXP_003633543    TLSFKEGDCG LQSASGAIDP RCEMDLPCFH PHVGSSSQAS ISGSGKMVVS
TC0010G00260      ATILKEGDCG VQSTDESIGL QCQTDLPCFH PHAGSSSQAS IGGSEKMNIS
MA04G10420        GCLQC-DADG QCIS------ ---------- QNLAAI---D W---EGSHVP
ZM08G18670        GSVECGADPA LRFD------ ---------- GSSEVVGGGG W---EKSSSP
ZM06G27420        GSVAC-ADPA LCFN------ ---------- GNSEVG--GG W---EESSRP
BD2G21197         MPLQCSVGSV VCSDR----- ---------- PALCFNGNSE AGAWEESSRP
BD2G52590         KPCAFGTD-- ---------- ---------- ----HAPCTG EVAAAAIRDV
Bradi4g43322      GDALSCDRVV QCLA------ ---------- -GKSELGMNS NCGPVALNCD
PPE_002G00940     CFCSPVGPSM QASI------ ---------- -STSAKAMVP VGGNCQPAVI
SB09G024290       -GSVACADPA LCFN------ ---------- -GNSEVGGCW EESSRPAVAD
OS05G41540        GSVACTDPGM CFNGNS---- ---------- -EIGGVWEEC SRPVGADRMI
OS01G58760        GSVTCTDHSP CFNTSTE--- ---------- V---AVREES SRPTIVDCGI
SB03G037300       GSVICTDPTL CFVNDDA--- ---------- EVAVPAREES SAPTNFSFEM
BR09G33550        SGFVFKEG-- ---------- ---------- ---DVSLL-- -SVCV-GYIY
CRU_005G17180     SGLVFKED-- ---------- ---------- ---GCSVATS NIICEAARVE
TBA00029289       ---------- ---------- ---------- ---------- ----------
Consistency       4333323322 1121000000 0000000000 0112232223 3222323323

                  .........260.........270.........280.........290.........300
AT3G51960         TLHDPIQSFV PQPPPFSR-- ---------- ---------- ----------
AL5G21660         TLHDPIHSFV PQSPPFSH-- ---------- ---------- ----------
BR04G06070        TLHEPIHSFV PHSPPFSR-- ---------- ---------- ----------
TP5G10310         ALHEPIHSFF PHSPPFSR-- ---------- ---------- ----------
PTA00035910       RCETDVPCFH P---PF---- ---------- ---------- ----------
MA04G00320        VVDCQIDPNG DDRAELGDR- ---------- ---------- ----------
ATR_00159G00320   ISCQLANSDI ASRAFSSAET QLDSLIPANE MGISGSLVAS ISQAE-----
VVXP_003633543    WEGNCQPAVV DCRGNINDMV SAEGHT---- MDTVEALVSS ASQAE-----
TC0010G00260      WEGNCQPAIV NCQANINQ-- ---------- MEVLKDTT-- ----------
```

Supplementary Figure S3. (cont.)

```
MA04G10420       AITNCQINPS GDIIMRQKPE I-AEAVNS-- MNVVGSLISS ASQTE-----
ZM08G18670       QVADCRID-E GGGGMSRELD VLEGLRHS-- IDAGATFVSS DSLAERSCLP
ZM06G27420       AAADCRIDEV RGMSREIDVP E--GLRHS-- MDVVASFVSS DPLAE-----
BD2G21197        AAAGCRFEED GNGGVARE-- ---------- IDVLEQVHS- MDVADLCFHS
BD2G52590        REVDCGIDES GI--ASVE-- ---------- ADLPELADSV MDADELCCLI
Bradi4g43322     ISPDSGQNLG TPYQAGCI-- ---------- ELRYQPGFRA EPGDALSSGL
PPE_002G00940    DCQANTNEMA SAVQNST--- ---------- --HSRN-LGI IGNRS-----
SB09G024290      VSEGL-RNSM DVVASFV--- ---------- --SSDP-LAE ----------
OS05G41540       DKDGSMSQEI DIPGPVHSIS ---------- MDVVGSLVTS ASLSE-----
OS01G58760       DGTGIISHEL DIPKMVNS-- ---------- VDVIPSFVNS ASLTE-----
SB03G037300      DESDSISREF DIPEVVNS-- ---------- MDAAASLVNS ASMAE-----
BR09G33550       DIGDK----- ---------- ---------- ---------- ----------
CRU_005G17180    CEEG------ ---------- ---------- ---------- ----------
TBA00029289      ---------- ---------- ---------- ---------- ----------
Consistency      3334222322 3112232200 0000000000 1111111111 1111100000

                 .
AT3G51960        -
AL5G21660        -
BR04G06070       -
TP5G10310        -
PTA00035910      -
MA04G00320       -
ATR_00159G00320  -
VVXP_003633543   -
TC0010G00260     -
MA04G10420       -
ZM08G18670       G
ZM06G27420       -
BD2G21197        -
BD2G52590        G
Bradi4g43322     H
PPE_002G00940    -
SB09G024290      -
OS05G41540       -
OS01G58760       -
SB03G037300      -
BR09G33550       -
CRU_005G17180    -
TBA00029289      -
Consistency      0
```

Supplementary Figure S4. Synteny analysis in Eudicot F-bZIPs. (**a**) Within-genome synteny analysis in *A. thaliana* F-bZIPs indicates presence of a syntenic block containing *AtbZIP19* and *AtbZIP23*. (**b**) Between-genome pairwise analysis of the same syntenic block across several Eudicot species was used to calculate the ratio of nonsynonymous per synonymous substitutions (Ka/Ks). Syntenic data was retrieved from the Plant Genomic Duplication Database (http://chibba.agtec.uga.edu/duplication/; see Methods section for details). *At*, *Arabidopsis thaliana*; *Cp*, *Carica papaya*; *Tc*, *Theobroma cacao*; *Ppe*, *Prunus persica*; *Mt*, *Medicago truncatula*; *Vv*, *Vitis vinifera*; *Sl*, *Solanum lycopersicum*.

a

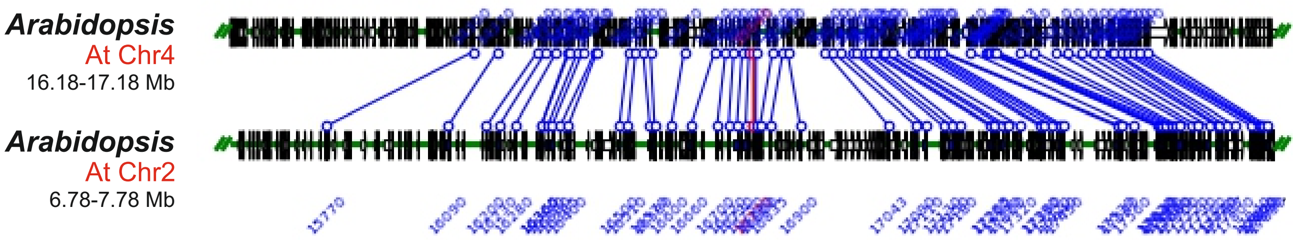

b

	Gene ID	*At* *AT4G35040*	*At* *AT2G16770*	*Cp* *evm.TU.supercontig_37.130*	*Tc* *Thecc1EG006625*	*Ppe* *ppa009913m*	*Mt* *Medtr4g073100*	*Vv* *GSVIVT01024024001*	*Sl* *Solyc01g111580.2*
At	*AT4G35040*		0.283	0.185	0.192	0.126	0.149	ND	0.150
At	*AT2G16770*	0.283		0.147	0.192	0.148	ND	0.143	0.148
Cp	*evm.TU.supercontig_37.130*	0.185	0.147		0.127	0.120	0.097	0.104	0.143
Tc	*Thecc1EG006625*	0.192	0.192	0.127		0.133	0.105	0.159	0.153
Ppe	*ppa009913m*	0.126	0.148	0.120	0.133		0.118	0.190	0.091
Mt	*Medtr4g073100*	0.149	ND	0.097	0.105	0.118		0.129	0.111
Vv	*GSVIVT01024024001*	ND	0.143	0.104	0.159	0.190	0.129		0.110
Sl	*Solyc01g111580.2*	0.150	0.148	0.143	0.153	0.091	0.111	0.110	

0 – 0.5

Supplementary Figure S5. Phylogenetic tree of ZIP family proteins in various plant species. ZIP orthologs were retrieved from the same set of representative plant species used in the F-bZIP phylogenetic analysis (see Methods section for details). The phylogenetic tree was constructed using maximum-likelihood and bootstrap analysis with 1000 replicates. The ZIP proteins highlighted in red represent the clade that incorporates orthologs of *A. thaliana* ZIP4, ZIP9 and IRT3.

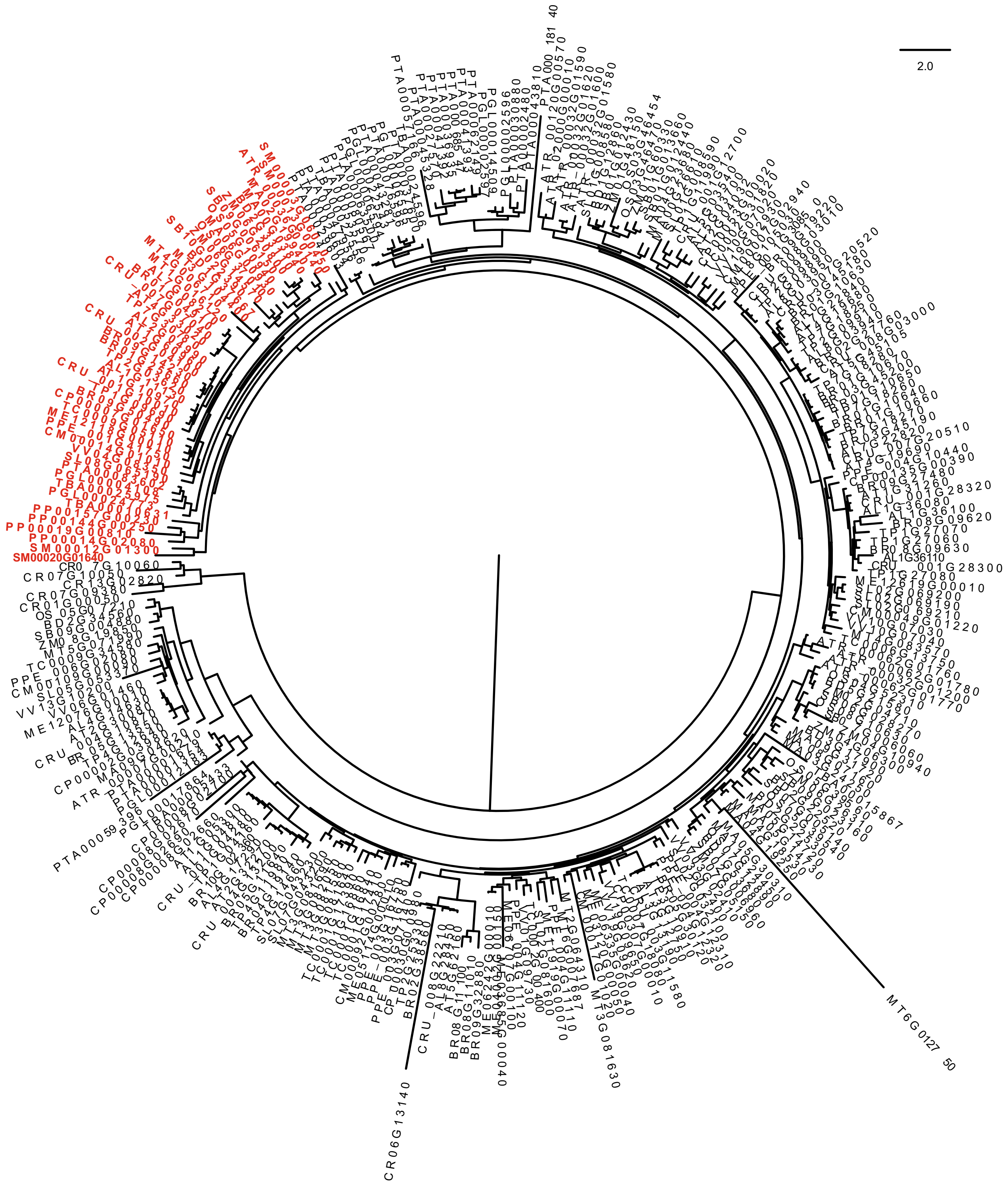

Supplementary Figure S6. Sequence logo of the *ZDRE* motif. The search for the *ZDRE cis*-element in the promoter region of the *ZIP* genes orthologs of *A. thaliana ZIP4/9/IRT3* and *ZIP2/ZIP11* groups was performed in the Multiple Em for Motif Elicitation (MEME, v4.11.2; http://meme-suite.org/tools/meme). The sequence logo of the detected *ZDRE* elements is represented.

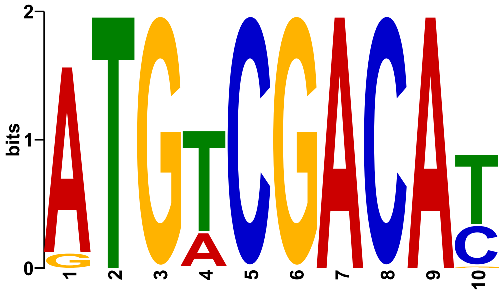

Supplementary Table S1. Plant F-bZIPs sequence IDs. The ID codes were obtained from Plaza (http://bioinformatics.psb.ugent.be/plaza/), Phytozome (https://phytozome.jgi.doe.gov/pz/) and NCBI (https://www.ncbi.nlm.nih.gov/) databases.

Plant species	Plaza ID	Phytozome ID	NCBI ID	SpotID
Angiosperms - Eudicots				
Arabidopsis thaliana	AT4G35040	AT4G35040		253171_at
	AT2G16770	AT2G16770		
	AT3G51960	AT3G51960		252083_at
Arabidopsis lyrata	AL7G05920	944394		
	AL3G37390	931513		
	AL5G21660	485555		
Capsella rubella	CRU_007G04950	Carubv10005538m.g		
	CRU_003G29440	Carubv10014436m.g		
	CRU_005G17180	Carubv10018125m.g		
Brassica rapa	BR08G13570	Brara.H01165		
	BR01G03220	Brara.A00325		
	BR09G08750	Brara.C04162		
	BR03G40060	Brara.I01006		
	BR04G06070	Brara.D00620		
	BR09G33550			
Thellungiella parvula	TP7G32810			
	TP3G29960			
	TP5G10310			
Carica papaya	CP00037G01300	evm.TU.supercontig_37.136		
Theobroma cacao	TC0002G05770	Thecc1EG006625		
	TC0010G00260	Thecc1EG042360		
Cucumis melo	CM00045G01020			
	CM00019G02650			
Prunus persica	PPE_008G18270	Prupe.8G165400		
	PPE_002G00940	Prupe.2G025800		
Medicago truncatula	MT4G073100	Medtr4g073100		Mtr.47031.1.S1_at
Manihot esculenta	ME01604G00020	Manes.11G093900		
	ME11821G00090	Manes.04G075800		
Vitis vinifera	VV03G04720	GSVIVG01024024001	XP_002278836.1	
	VV13G01230	GSVIVG01001468001	XP_003633543.1	
Solanum lycopersicum	SL01G111580	Solyc01g111580.2		LesAffx.46727.1.S1_at
	SL10G055550	Solyc10g055550.1		
Angiosperms - Monocots				
Zea mays	ZM05G15830	GRMZM2G000171		GRMZM2G000171
	ZM06G10070	GRMZM2G055413		GRMZM2G055413
	ZM06G27420	GRMZM2G175870		GRMZM2G175870
	ZM08G18670	GRMZM2G033230		GRMZM2G033230
Sorghum bicolor	SB10G030250	Sobic.010G267500		
	SB09G024290	Sobic.009G182800		
	SB03G037300	Sobic.003G327000		

Supplementary Table S1. (cont.)

Oryza sativa	OS06G50310	LOC_Os06g50310	Os.5399.1.S1_at
	OS05G41540	LOC_Os05g41540	OsAffx.23844.1.S1_at
	OS01G58760	LOC_Os01g58760	Os.26821.1.S1_at
Brachypodium distachyon	BD1G30140	Bradi1g30140	
	BD2G21197	Bradi2g21197	
	BD2G52590	Bradi2g52590	
		Bradi4g43322	
Musa acuminata	MA01G15430	GSMUA_Achr1G14830_001	
	MA03G00680	GSMUA_Achr3G00670_001	
	MA07G03920	GSMUA_Achr7G03840_001	
	MA01G27530	GSMUA_Achr1G26460_001	
	MA04G00320	GSMUA_Achr4G00320_001	
	MA04G10420	GSMUA_Achr4G10150_001	
Angiosperms - Amborellale			
Amborella trichopoda	ATR_00152G00060	AmTr_v1.0_scaffold00152	
	ATR_00159G00320	AmTr_v1.0_scaffold00159	
Gymnosperms			
Picea glauca	PGL00007063		
	PGL00017522		
Pinus taeda	PTA00032252		
	PTA00035910		
	PTA00084128		
Taxus baccata	TBA00003354		
	TBA00005280		
	TBA00029289		
Pteridophyte			
Selaginella moellendorffii	SM00011G03560	Sm409920	
		Sm414462	
Bryophyte			
Physcomitrella patens	PP00034G00570	Pp3c14_19470	Phypa_26362
		Pp3c17_21620	
	PP00068G00380		

Supplementary Table S2. Differentially expressed genes detected by comparative microarray analysis. *A. thaliana* wild-type (WT) and *bzip19/23* double mutant plants root and shoot transcriptome were compared. Three zinc treatments were applied; zinc deficiency, Zn- (0.05 µM), zinc sufficiency, control (2 µM), and zinc excess, Zn+ (25 µM). Genes significantly down-regulated and up-regulated are highlighted in green and red, respectively.

				Roots						Shoots					
				***bzip19/23* Zn- vs WT Zn-**		***bzip19/23* Cont. vs WT Cont.**		***bzip19/23* Zn+ vs WT Zn+**		***bzip19/23* Zn- vs WT Zn-**		***bzip19/23* Cont. vs WT Cont.**		***bzip19/23* Zn+ vs WT Zn+**	
**SpotID**	**AGI code**	**Gene name**	**Annotation**	**Fold-change**	**Adj. *p* -value**	**Fold-change**	**Adj. *p* -value**	**Fold-change**	**Adj. *p* -value**	**Fold-change**	**Adj. *p* -value**	**Fold-change**	**Adj. *p* -value**	**Fold-change**	**Adj. *p* -value**
266336_at	At2g32270	*ZIP3*	Zinc transporter 3 precursor	-4,719919592	7,43E-10	-3,869930894	1,11E-06	-1,70548412	0,009141345	-0,319898001	0,175355275	-0,033586133	0,960791947	-0,015929075	0,978947284
251438_s_at	At3g59930 At5g33355		Defensin-like (DEFL) family protein	-5,329158371	3,89E-09	-3,16343902	3,93E-05	-0,201370689	0,83425765	-1,094136708	0,108264181	-0,254100838	0,669571886	-0,188044045	0,65644854
249780_at	At5g24240		Phosphatidylinositol 3- and 4-kinase; Ubiquitin family protein	-3,219442095	1,18E-08	-3,289096261	1,15E-07	-3,759786657	4,33E-06	-3,761528377	0,000320681	-3,449344927	0,000125543	-3,659694472	1,76E-05
253171_at	At4g35040	*bZIP19*	Basic-leucine zipper (bZIP) transcription factor family protein	-5,257504751	1,38E-08	-5,403547884	1,07E-08	-5,193930163	8,68E-08	-4,253817406	1,33E-05	-4,295625721	1,97E-07	-4,03224881	7,71E-05
260462_at	At1g10970	*ZIP4*	Zinc transporter 4 precursor	-2,79745044	6,84E-08	-1,240196852	0,002429716	-0,41333559	0,285564509	-3,137703087	0,002822558	-0,739469257	0,256087157	0,036309287	0,923960238
264574_at	At1g05300	*ZIP5*	Zinc transporter 5 precursor	-3,143964127	3,28E-07	-1,691571709	5,32E-06	-0,875482643	0,113822101	-1,284784368	0,076590516	-0,501488967	0,333800625	0,057658905	0,863706421
253413_at	At4g33020	*ZIP9*	ZIP metal ion transporter family	-1,724462225	7,90E-06	-0,58221216	0,402477193	-0,372814702	0,422264699	-0,16947251	0,504020821	-0,104007286	0,899718042	0,073191434	0,786549577
249127_at	At5g43500	*ARP9*	Actin-related protein 9	-1,20811408	1,59E-05	-1,217562719	0,000531188	-1,325449709	0,001592124	-0,954996231	0,076590516	-0,823346477	0,333800625	-1,212284596	0,030856981
257715_at	At3g12750	*ZIP1*	Zinc transporter 1 precursor	-1,697839322	6,04E-05	-0,922558562	0,05043719	-0,513746803	0,285564509	-0,876013665	0,076590516	-0,351306674	0,367603658	-0,131034493	0,729215007
248499_at	At5g50400	*PAP27*	Purple acid phosphatase 27	-1,329185905	0,000168951	-0,714786381	0,00212136	-0,267757868	0,296639232	-1,627417529	0,00368188	-0,554090682	0,333800625	-0,061314274	0,826891402
259632_at	At1g56430	*NAS4*	Nicotianamine synthase 4	-1,159697981	0,001127539	-0,604248232	0,402668144	-0,475655732	0,433484592	-1,10331998	0,0838519	-0,212110755	0,654240498	-0,569560427	0,320397593
258646_at	At3g08040	*FRD3, MAN1*	MATE efflux family protein	-0,831965264	0,005835863	-0,448869357	0,584333376	-0,39429801	0,443716788	-0,10848961	0,680431479	-0,026092614	0,962781571	-0,041466254	0,897859749
255940_at	At1g20380		Prolyl oligopeptidase family protein	-0,865824718	0,006433499	-0,551728289	0,402477193	-0,139292285	0,62450724	-0,109760071	0,665004552	0,00955241	0,99128089	0,047806547	0,910686949
266783_at	At2g29130	*LAC2*	Laccase 2	0,819030865	0,010804473	-0,02809621	0,965491452	-0,121127612	0,65634552	0,009804263	0,975991483	0,035149573	0,92404719	0,043097889	0,919388431
259661_at	At1g55265		Protein of unknown function, DUF538	-0,444313384	0,019030699	0,028396501	0,945037612	0,050744309	0,905056996	-0,195343855	0,618000916	-0,144418645	0,894432432	0,287318052	0,376977494
266695_at	At2g19810	*OZF1, TZF2*	CCCH-type zinc finger family protein	0,490784458	0,027706794	0,163364745	0,772035217	0,118847507	0,785957277	0,083926767	0,910310424	-0,096188573	0,78413887	0,157886814	0,856547984
248048_at	At5g56080	*NAS2*	Nicotianamine synthase 2	-1,612301169	0,028205218	-0,657484969	0,689307459	-0,914586503	0,400837989	0,006165349	0,987328706	0,017286356	0,968087944	-0,225130877	0,455711204
255129_at	At4g08290	*UMAMIT20*	Nodulin MtN21 /EamA-like transporter family protein	0,613979485	0,028205218	0,703680979	0,431777745	0,704027827	0,461833575	0,085334775	0,76593944	0,095826536	0,739053963	-0,19439425	0,457255432
255252_at	At4g04990		Protein of unknown function, DUF761	-0,621091958	0,028205218	0,046995548	0,95183142	-0,12553307	0,853682475	-0,107620838	0,528373217	-0,01521613	0,976840574	0,067146283	0,853186542
260287_at	At1g80440	*KFB20, KMD1*	Galactose oxidase/kelch repeat superfamily protein	0,636411614	0,028205218	0,589777526	0,498722244	0,181637364	0,710717261	1,050396579	0,096195161	0,434412174	0,333800625	0,488380726	0,53895122
260973_at	At1g53490	*HEI10*	RING/U-box superfamily protein	-0,568666481	0,028205218	-0,384948666	0,402477193	-0,329965051	0,323791094	-0,268831066	0,310078573	-0,143943783	0,646196206	-0,237121454	0,43908116
260135_at	At1g66400	*CML23*	Calmodulin like 23	-0,45630379	0,031738875	-0,094390277	0,82394078	0,222071092	0,700205092	-0,182656146	0,434551752	0,062775466	0,898082506	0,181319145	0,611353239
257176_s_at	At3g23510 At3g23530		Cyclopropane-fatty-acyl-phospholipid synthase	0,496313772	0,036861546	0,272836801	0,441544074	0,322994032	0,456909321	0,426263165	0,252402248	0,181861702	0,659401767	-0,216625652	0,592760117
264908_at	At2g17440	*PIRL5*	Plant intracellular ras group-related LRR 5	0,456821375	0,036861546	0,356139803	0,419027491	0,123674602	0,775205715	0,613583226	0,105337508	0,078967975	0,807126347	-0,010553353	0,985779297
256438_s_at	At2g40205 At3g08520 At3g11120 At3g56020		Ribosomal protein L41 family	-0,61938465	0,036909912	-0,264889705	0,533946553	0,939434996	0,467279858	-0,683943373	0,298528864	-0,411985841	0,681386916	-0,064065341	0,942886967
259718_at	At1g61040	*VIP5*	Plus-3 domain-containing protein	0,429302315	0,038174253	0,336736144	0,409624883	0,010722304	0,974959666	0,117117347	0,681604659	-0,003477666	0,997688526	-0,013257889	0,975855023
249527_at	At5g38710		Methylenetetrahydrofolate reductase family protein	-0,454416181	0,041594456	-0,796390931	0,268525436	-0,555296165	0,285564509	0,515330249	0,20880689	0,324412552	0,540080378	0,32323626	0,345187787
264572_at	264572_at			-0,5137905	0,041594456	-0,083180983	0,84417565	0,148675084	0,562789604	-0,451728806	0,273088162	-0,099224874	0,765862664	-0,087683889	0,774371495
266209_at	At2g27550	*ATC*	Centroradialis	-0,680758398	0,117112693	-1,205682124	0,011360928	-0,798975889	0,357972155	0,007622768	0,976807143	-0,081020996	0,900213211	0,124012288	0,721363897
246530_at	At5g15725	*GLV9*	Unknown protein	-0,379234245	0,39668578	0,190379121	0,653981894	0,690933368	0,018836692	-0,022895385	0,91222586	-0,043190876	0,942995559	-0,132353133	0,686815976
263948_at	At2g35980	*NHL10, YLS9*	Late embryogenesis abundant (LEA) hydroxyproline-rich glycoprotein family	0,550699015	0,170502819	0,285910115	0,459435928	-0,276150563	0,372766652	1,027554832	0,047710843	0,365405777	0,633960364	0,408065976	0,83777522
262072_at	At1g59590	*ZCF37*		0,140191089	0,405096688	0,292658442	0,424714972	0,204112689	0,490289106	0,839030599	0,047710843	0,338272006	0,456635534	0,450365891	0,559612526
262177_at	At1g74710	*EDS16, ICS1, SID2*	ADC synthase superfamily protein	-0,215161251	0,49788671	-0,084799435	0,793682768	0,102707752	0,575634634	1,032310548	0,047710843	0,497148523	0,354535228	0,304703404	0,851052756
247577_at	At5g61290		Flavin-binding monooxygenase family protein	0,065989949	0,765924962	0,104848786	0,633485989	-0,125806706	0,631436989	-0,803665834	0,047710843	-0,21897172	0,707504555	0,278137295	0,71072368
251970_at	At3g53150	*UGT73D1*	UDP-glucosyl transferase 73D1	0,147242458	0,439899189	0,11469375	0,711809398	-0,027083428	0,910037333	1,505390238	0,047710843	0,46716372	0,378257641	0,446942626	0,479842349
261763_at	At1g15520	*ABCG40, PDR12*	Pleiotropic drug resistance 12	-0,1442611	0,428994636	-0,061900429	0,901948591	-0,016576262	0,972483955	0,946680082	0,047710843	0,468262285	0,550668675	0,157535432	0,832093331
245035_at	At2g26400	*ARD3*	Acireductone dioxygenase 3	-0,081133266	0,940119249	-0,422393411	0,468578292	0,028688491	0,981626789	1,171575307	0,047710843	0,499757461	0,470884408	0,536061096	0,686701402
264645_at	At1g08940		Phosphoglycerate mutase family protein	0,153887736	0,383785848	-0,045647137	0,854755118	-0,2047231	0,526785175	0,869188132	0,05449961	0,773768884	0,042997492	1,235246312	0,001794964
247814_at	At5g58310	*MES18*	Methyl esterase 18	-0,107036278	0,456721179	0,008818939	0,985690161	-0,156818819	0,603319394	-1,375159442	0,102212136	-0,890389261	0,042997492	-0,17121739	0,747210201
248321_at	At5g52740		Copper transport protein family	-0,041988554	0,777477824	-0,126891604	0,533984697	-0,086890297	0,650036817	-0,05048217	0,963121038	-0,263141444	0,873383851	0,737110276	0,030856981

Supplementary Table S3. Gene ontology (GO) enriched categories for Molecular Function of down-regulated genes in *Atbzip19/23* double mutant roots in response to zinc deficiency. For each category, the adjusted *p*-value is indicated between brackets. Gene annotations are based on the BAR database (http://bar.utoronto.ca/).

Spot ID	AGI code	Gene name	Gene annotation	Fold-change (Log2)	Adj. *p*-value
**GO:** ***Cation transmembrane transporter activity*** (*p*-value 9.0983E-9)					
266336_at	At2g32270	*ZIP3*	Zinc transporter 3 precursor	-4,72	7,43E-10
264574_at	At1g05300	*ZIP5*	Zinc transporter 5 precursor	-3,14	3,28E-07
260462_at	At1g10970	*ZIP4*	Zinc transporter 4 precursor	-2,80	6,84E-08
253413_at	At4g33020	*ZIP9*	ZIP metal ion transporter family	-1,72	7,90E-06
257715_at	At3g12750	*ZIP1*	Zinc transporter 1	-1,70	6,04E-05
**GO:** ***Nicotianamine synthase activity*** (*p*-value 3.3436E-5)					
248048_at	At5g56080	*NAS2*	Nicotianamine synthase 2	-1,61	0,02820522
259632_at	At1g56430	*NAS4*	Nicotianamine synthase 4	-1,16	0,00112754

Supplementary Table S4. Annotation of *ZIP* gene orthologs of *A. thaliana ZIP4/9/IRT3*, sequence IDs and presence of *ZDRE cis*-elements in the promoter region. The database for protein and promoter search was the Plaza database (http://bioinformatics.psb.ugent.be/plaza/). The search for *ZDRE* motif (RTGTCGACAY) in the promoter region was performed in Motif Alignment & Search Tool (MAST v4.11.2; http://meme-suite.org/tools/mast). Position for each *ZDRE cis*-element refers to nucleotide distance to the +1 nucleotide of the translation start codon.

Plant species	Plaza gene ID	No. motifs	Motif	*p*-value	Position	No. mismatches
Angiosperms - Eudicots						
Arabidopsis thaliana	AT1G10970 (*ZIP4*)	2	**ATGTCGACAT**	7,30E-06	-246	
			**ATGTCGACAC**	7,30E-06	-118	
	AT1G60960 (*IRT3*)	2	**ATGTCGACAT**	7,30E-06	-264	
			**ATGTCGACAT**	7,30E-06	-166	
	AT4G33020 (*ZIP9*)	1	**ATGACGACAT**	7,30E-06	-579	1
Arabidopsis lyrata	AL1G11130	2	**ATGTCGACAT**	7,30E-06	-235	
			**ATGTCGACAC**	7,30E-06	-116	
	AL2G04990	2	**ATGTCGACAT**	7,30E-06	-268	
			**ATGTCGACAT**	7,30E-06	-160	
	AL7G08270	1	**ATGACGACAT**	7,30E-06	-661	1
Capsella rubella	CRU_001G10010	2	**ATGTCGACAT**	7,30E-06	-256	
			**ATGTCGACAC**	7,30E-06	-137	
	CRU_002G04100	2	**ATGTCGACAT**	7,30E-06	-249	
			**ATGTCGACAT**	7,30E-06	-141	
	CRU_007G07040	1	**ATGACGACAT**	7,30E-06	-612	1
Brassica rapa	BR01G04910	0				
	BR01G25660	2	**ATGTCGACAT**	7,30E-06	-657	
			**ATGTCGACAC**	7,30E-06	-552	
	BR09G14230	2	**ATGTCGACAT**	7,30E-06	-740	
			**ATGTCGACAT**	7,30E-06	-632	
	BR09G50490	2	**ATGTCGACAT**	7,30E-06	-768	
			**ATGTCGACAC**	7,30E-06	-661	
Thellungiella parvula	TP1G09690	2	**ATGTCGACAT**	7,30E-06	-574	
			**ATGTCGACAC**		-481	
	TP2G03620	2	**ATGTCGACAT**	7,30E-06	-418	
			**ATGTCGACAT**	7,30E-06	-300	
	TP7G30780	1	**ATGACGACAT**	7,30E-06	-563	1
Carica papaya	CP00007G01650	2	**ATGTCGACAC**	7,30E-06	-947	
			**ATGTCGACAC**	7,30E-06	-860	
Theobroma cacao	TC0009G05110	2	**ATGTCGACAT**	7,30E-06	-453	
			**ATGTCGACAC**	7,30E-06	-367	
Cucumis melo	CM00014G01010	2	**GTGTCGACAC**	7,30E-06	-321	
			**ATGTCGACAC**	7,30E-06	-235	
Prunus persica	PPE_001G41750	2	**GTGTCGACAC**	7,30E-06	-632	
			**ATGTCGACAC**	7,30E-06	-545	

Supplementary Table S4. (cont.)

Medicago truncatula	MT1G016120	1	ATGTCGACAT	7,30E-06	-407	
	MT3G104400	2	ATGTCGACAC	7,30E-06	-537	
			ATGTCGACAC	7,30E-06	-452	
	MT4G006710	0				
Manihot esculenta	ME12118G00010	3	ATGTCGTCAT	7,30E-06	-401	1
			GTGTCGACAT	7,30E-06	-302	
			ATGTCGACAC	7,30E-06	-215	
Vitis vinífera	VV04G08350	2	GTGTCGACAC	7,30E-06	-227	
			ATGTCGACAT	7,30E-06	-141	
Solanum lycopersicum	SL08G065190	2	ATGTCGACAG	2,80E-05	-279	1
			ATGTCGACAT	7,30E-06	-203	
Angiosperms - Monocots						
Zea mays	ZM06G14570	1	GTGTCGACAT	7,30E-06	-381	
	ZM06G20800	2	ATGTCGACAT	7,30E-06	-650	
			ATGACGACAT	7,30E-06	-211	1
Sorghum bicolor	SB09G006150	2	ATGTCGACAT	7,30E-06	-636	
			ATGACGACAT	7,30E-06	-232	1
	SB10G022390	2	GTGTCGACAT	7,30E-06	-719	
			ATGACGACAT	7,30E-06	-510	1
Oryza sativa	OS05G10940	2	ATGTCGACAT	7,30E-06	-641	
			GTGACGACAT	7,30E-06	-298	1
	OS06G37010	3	GTGTCGACAC	7,30E-06	-714	
			ATGACGACAT	7,30E-06	-495	1
			ATGACGACAT	7,30E-06	-465	1
Brachypodium distachyon	BD1G37667	2	GTGTCGACAT	7,30E-06	-871	
			ATGACGACAT	7,30E-06	-665	1
	BD2G33110	2	ATGTCGACAT	7,30E-06	-650	
			ATGACGACAT	7,30E-06	-200	1
Musa acuminata	MA00G03840	2	ATGTCGACAT	7,30E-06	-563	
			ATGTCGACAT	7,30E-06	-390	
	MA02G19940	4	ATGTCGACAT	7,30E-06	-625	
			GTGTCGCCAT	2,80E-05	-566	1
			ATGTCGACAT	7,30E-06	-555	
			ATGACGACAT	7,30E-06	-368	1
	MA06G00930	2	GTGTCGACAT	7,30E-06	-600	
			ATGACGACAT	7,30E-06	-357	1
Angiosperms - Amborellale						
Amborella trichopoda	ATR_00036G00140	2	ATGTCGTCAT	7,30E-06	-1184	1
			GTGTCGACAC	7,30E-06	-306	
Gymnosperms						
Picea glauca	PGL00004108					
	PGL00024773	(incomplete sequence)				

Supplementary Table S4. (cont.)

Pinus taeda	PTA00083602	2	CTGTCGACAT	2,80E-05	-784	1
			ATGACGACAT	7,30E-06	-204	1
Taxus baccata	TBA00010631	(incomplete sequence)				
	TBA00025975	(incomplete sequence)				
Pteridophyte						
Selaginella moellendorffii	SM00003G06040	1	ATGTCGACAC	7,30E-06	-309	
	SM00012G01300	1	ATGTCGACAT	7,30E-06	-226	
	SM00013G00450	0				
	SM00020G01640	0				
Bryophyte						
Physcomitrella patens	PP00014G02080	1	ATGACGACAC	7,30E-06	-718	1
	PP00019G00810	0				
	PP00144G00250	1	ATGACGACAT	7,30E-06	-955	1
	PP00157G00230	0				

Supplementary Table S5. Annotation of *ZIP* gene orthologs of *A. thaliana ZIP2/11*, sequence IDs and presence of *ZDRE cis*-elements in the promoter region. The database for protein and promoter search was the Plaza database (http://bioinformatics.psb.ugent.be/plaza/). The search for *ZDRE* motif (RTGTCGACAY) in the promoter region was performed in Motif Alignment & Search Tool (MAST v4.11.2; http://meme-suite.org/tools/mast). Position for each *ZDRE cis*-element refers to nucleotide distance to the +1 nucleotide of the translation start codon.

Plant species	Plaza gene ID	No. motifs	Motif	*p*-value	Position	No. mismatches
Angiosperms - Eudicots						
Arabidopsis thaliana	AT5G59520 (*ZIP2*)					
	AT1G55910 (*ZIP11*)	1	**GTGTCGTAAT**	9,90E-05	-153	2
Angiosperms - Monocots						
Oryza sativa	OS01G74110					
	OS03G29850					
	OS08G42150					
	OS08G42170					
Angiosperms - Amborellale						
Amborella trichopoda	ATR_00012G02350					
	ATR_00028G00060					
Gymnosperm						
Picea glauca	PGL00001228	(incomplete sequence)				
	PGL00017583					
	PGL00017781					
	PGL00027832	(incomplete sequence)				
	PGL00028376	(incomplete sequence)				
Bryophyte						
Physcomitrella patens	PP00017G01250					
	PP00123G00560					
	PP00144G00590	1	**ATGTTGTCAT**	9,90E-05	-182	2
	PP00225G00080	1	**GTGTCGTCTT**	9,90E-05	-1441	2

Supplementary Table S6. Primers used for genotyping transgenic *A. thaliana pAtZIP4::GUS* lines in the double mutant *Atbzip19/23* background.

Primer name	Sequence 5’-3’	Employment
LBb1.3	ATTTTGCCGATTTCGGAAC	Genotyping SALK T-DNA insertion lines
bZIP19 RP1	ACGATGCCATCTGTTTAGTGC	Genotyping *Atbzip19-1* (SALK_144252)
bZIP19 LP1	ATTGACGTTGCTGAATGATCC	Genotyping *Atbzip19-1* (SALK_144252)
bZIP23 LP1	TTTTTCATTCACCATTTTTCATTG	Genotyping *Atbzip23-1* (SALK_045200)
bZIP23 RP1	TCATAACCTCATCCTCCAACG	Genotyping *Atbzip23-1* (SALK_045200)
pAtZIP4::GUS F1	TACTGATTTGCCTCTTTTGGC	Genotyping *pAtZIP4::GUS* transgenic lines
pAtZIP4::GUS R1	TTATCGAATCCTTTGCCACG	Genotyping *pAtZIP4::GUS* transgenic lines
